# Deorphanizing solute carriers in *Saccharomyces cerevisiae* for secondary uptake of xenobiotic compounds

**DOI:** 10.3389/fmicb.2024.1376653

**Published:** 2024-04-12

**Authors:** Iben Møller-Hansen, Javier Sáez-Sáez, Steven A. van der Hoek, Jane D. Dyekjær, Hanne B. Christensen, Marina Wright Muelas, Steve O’Hagan, Douglas B. Kell, Irina Borodina

**Affiliations:** ^1^The Novo Nordisk Foundation Center for Biosustainability, Technical University of Denmark, DK-2800 Kgs, Lyngby, Denmark; ^2^Department of Biochemistry and Systems Biology, Institute of Systems, Molecular and Integrative Biology, University of Liverpool, Liverpool, United Kingdom; ^3^Department of Chemistry, Manchester Institute of Biotechnology, The University of Manchester, Manchester, United Kingdom

**Keywords:** small molecule transporters, xenobiotic substrates, metabolite transport, cell factories, transporter protein, exometabolome, metabolic engineering, substrate specificity

## Abstract

The exchange of small molecules between the cell and the environment happens through transporter proteins. Besides nutrients and native metabolic products, xenobiotic molecules are also transported, however it is not well understood which transporters are involved. In this study, by combining exo-metabolome screening in yeast with transporter characterization in *Xenopus* oocytes, we mapped the activity of 30 yeast transporters toward six small non-toxic substrates. Firstly, using LC–MS, we determined 385 compounds from a chemical library that were imported and exported by *S. cerevisiae*. Of the 385 compounds transported by yeast, we selected six compounds (*viz.* sn-glycero-3-phosphocholine, 2,5-furandicarboxylic acid, 2-methylpyrazine, cefadroxil, acrylic acid, 2-benzoxazolol) for characterization against 30 *S. cerevisiae* xenobiotic transport proteins expressed in *Xenopus* oocytes. The compounds were selected to represent a diverse set of chemicals with a broad interest in applied microbiology. Twenty transporters showed activity toward one or more of the compounds. The tested transporter proteins were mostly promiscuous in equilibrative transport (i.e., facilitated diffusion). The compounds 2,5-furandicarboxylic acid, 2-methylpyrazine, cefadroxil, and sn-glycero-3-phosphocholine were transported equilibratively by transporters that could transport up to three of the compounds. In contrast, the compounds acrylic acid and 2-benzoxazolol, were strictly transported by dedicated transporters. The prevalence of promiscuous equilibrative transporters of non-native substrates has significant implications for strain development in biotechnology and offers an explanation as to why transporter engineering has been a challenge in metabolic engineering. The method described here can be generally applied to study the transport of other small non-toxic molecules. The yeast transporter library is available at AddGene (ID 79999).

## 1 Introduction

Transporter proteins mediate the import or export of nutrients, metabolic products, and other small molecules through equilibrative and concentrative transport across the membranes. Paradoxically, despite their potential applications in biotechnology and human medicine, transporter proteins are among the least characterized protein categories (César-Razquin et al., 2015). Only a handful of the 341 *S. cerevisiae* 288C transporters listed in the TransportDB database (Berninsone and Hirschberg, 2000; Baek et al., 2016; Baldi et al., 2021; Belew et al., 2022) have been experimentally characterized for substrate specificity. Recently, efforts have been made to deorphanize transporter proteins in humans (Superti-Furga et al., 2020) and plants (Nour-Eldin et al., 2006, 2012; Payne et al., 2017; Otterbach et al., 2019).

It has been suggested that the export of xenobiotic molecules occurs by ATP-binding cassette (ABC) transporters. The import of xenobiotics was proposed to happen via nutrient carriers (Almeida et al., 2021). While ABC transporters are unidirectional, carriers are hypothesized to be bidirectional depending on the concentration gradient of the co-substrate for anti-porters and symporters (Alberts et al., 2002), or by the concentration of the compound on both sides of the membrane for uniporters (Alberts et al., 2002; Zhang and Han, 2016). While substrate specificity is paramount to the function of a transporter protein, the mechanism of transport is equally fundamental for the application of transporter proteins (Darbani et al., 2019).

The current go-to approaches for high-throughput characterization of transporters in yeast (Giaever et al., 2002) and human cell lines (Girardi et al., 2020) relies on phenotypes that are easy to assess. This bias favors substrates that exhibit cellular toxicity or are fluorescent, excluding many non-toxic, non-fluorescent xenobiotic compounds important in biotechnology or health context. Another strategy uses untargeted metabolomics to determine differential uptake in cell lines differing in their expression levels of the target of interest (Gründemann et al., 2005; Wright Muelas et al., 2020).

In this study, we sought to experimentally assign novel substrates to transporters from transporter superfamilies with known capacity for xenobiotic substrates. To do so, we initially performed a global identification of potential substrates for yeast transporters from a chemical library. We used human blood serum as a low-cost ‘chemical library’ (Gründemann et al., 2005; Dunn et al., 2011; Wright Muelas et al., 2020) as it contains a wide array of compounds, from metabolites to xenobiotics, stemming from traces of dietary compounds and drugs (Dunn et al., 2011; Psychogios et al., 2011; Wright Muelas et al., 2020). Human blood serum is an ideal mix for assessing substrate specificity across different chemical classes. We used the yeast *S. cerevisiae*, as the screening organism, as it is a well-studied eukaryotic model organism with well-defined transporter proteins (Kell et al., 2011). In a previous effort, we identified the human and fungal genomes as having the same fraction of active transporters (Darbani et al., 2018), arguably suggesting that the results found in yeast are broadly applicable to eukaryotes. Besides, yeast has an extraordinary ability to import and export compounds found in its environment and hence possesses a repository of transporters for xenobiotics and metabolites.

Next, to address the question of which yeast transporters could transport the selected compounds and their mode of transport, we generated a dedicated library of full-length mRNA encoding multidrug or complex metabolite transporters from yeast. The candidate transporters were expressed in *Xenopus laevis* oocytes and tested for activity toward the target compounds.

*Xenopus* oocytes have unique features, such as low background transport activity, high capacity for correct transporter protein synthesis, and low codon bias (Romero et al., 1998). The low level of endogenous transporters makes it possible to determine transport activity toward a given substrate by extracting the whole *Xenopus* oocyte and analyzing the content by LC–MS. This facilitates identification of transporters for non-electrogenic substrates, making the method more broadly applicable than analysis by electrophysiology, as electrophysiology requires the generation of an electrical current for the detection of transport activity. Utilizing a similar methodology, *Xenopus* oocytes have been used successfully to identify plant metabolite transporters (Nour-Eldin et al., 2012; Tal et al., 2016; Jørgensen et al., 2017; Wulff et al., 2019; Xu et al., 2023) and characterize transporters for the natural compounds resveratrol, succinic acid, and malic acid (Darbani et al., 2019), as well as metabolomic profiling for determination of substrate specificity of transporter proteins (Fairweather et al., 2021).

## 2 Materials and methods

### 2.1 Buffers and reagents

Sodium acrylate (Sigma-Aldrich, Søborg, Denmark), sn-Glycero-3-phosphocholine, >98% (Santa Cruz Biotechnology, Inc., Heidelberg, Germany), cefadroxil 95–105%, (VWR, Søborg, Denmark), 2-methylpyrazine (VWR, Søborg, Denmark), and 2-benzoxazolinone, ≥98.0% (VWR, Søborg, Denmark), 2,5-furandicarboxylic acid (TCI Europe N. V., Zwijndrecht, Belgium) were used in the study. *Xenopus* oocytes were incubated in either HEPES-based Kulori buffer (90 mM NaCl, 1 mM KCl, 1 mM MgCl_2_, 1 mM CaCl_2_, 5 mM HEPES pH 7.4) supplemented with gentamycin (100 μg/mL), or MES-based Kulori buffer (90 mM NaCl, 1 mM KCl, 1 mM MgCl_2_, 1 mM CaCl_2_, 5 mM MES pH 5.0).

### 2.2 Strains, media and growth conditions

Yeast knockout mutant strains with BY4741 (MATa; *his3Δ1; leu2Δ0; met15Δ0; ura3Δ0*) background were purchased from Open Biosystems (catalog # YSC1053). For Growth Profiler 960 (Enzyscreen B.V., CR9001) measurements, yeast pre-cultures were inoculated in 2 mL synthetic dextrose medium (SD –Trp) (20 g/L glucose, 7 g/L yeast nitrogen base, 2 g/L histidine, 3 g/L leucine, 2 g/L methionine, 2 g/L uracil) in 15 mL growth tubes and grown for 24 h at 30°C and 250 rpm. Optical density at 600 nm (OD_600_) was then measured and adjusted to 0.01, in a 24 deep-well plate (Enzyscreen B.V., CR1424d), containing 2 mL SD –Trp supplemented with the indicated concentrations of additives. The cultures were grown for 72 h at 30°C with 300 rpm agitation and measurements acquired every 20 min.

### 2.3 Exometabolome assay

Pooled human blood serum, 0.1 μm filtered was purchased from BioIVT (West Sussex, United Kingdom). *S. cerevisiae* strain CEN.PK113-7D (MATa URA3 HIS3 LEU2 TRP1 MAL2-8c SUC2) was a gift from Peter Kötter (Goethe University, Frankfurt/Main, Germany).

An overnight preculture of CEN.PK113-7D was grown in YPD overnight, resuspended in YPD to an OD_600_ = 2. At OD_600_ = 8, 1 mL of culture was taken and the cells were centrifuged at 3,000 × *g* for 5 min. The cells were washed, and resuspended in MilliQ to a final OD_600_ of 2. 1 mL of the yeast solution was centrifuged at 3,000 × g for 5 min, and resuspended in 200 μL pooled human blood serum, to a final OD_600_ of 10. The experiment was performed in duplicate, with two independent biological replicates. After 30 min of incubation at 30°C in a shaking incubator at 250 rpm, the cells were collected by centrifugation at 3,000 x *g* for 5 min and 200 μL of the supernatant was mixed with 700 μL of ice-cold methanol. Filtered pooled human blood serum was used as control. The mixtures of supernatant or serum in methanol were mixed by vortexing, and cleared for protein precipitate by centrifugation. The supernatant was aliquoted and dried in a centrifugal evaporator with no temperature application. Prior to LC–MS analysis, samples were resuspended in LC–MS grade water.

All analyses were performed using an Ultimate 300 LC system (Thermo Fisher Scientific) using a HypersilGold a C18 2.1 mm x 100 mm, 1.9 μm column (Thermo Fisher Scientific), coupled to an Orbitrap Fusion Tribrid Mass Spectrometer (Thermo Fisher Scientific). Parameters for LC elution gradient and parameters for data acquisition on the MS/MS system are described in Wright Muelas et al., (2020).

The raw data files were imported into Progenesis QI (Non-linear Dynamics, Newcastle upon Tyne, United Kingdom) for processing. Peak alignment, picking and compound identification were performed using the software. Basic descriptive analysis, as well as statistical analysis to calculate fold changes in metabolites of yeast incubated pooled human blood serum versus normal pooled human blood serum was done in R (version 3.5.1). A threshold of Log_2_ fold change of compound levels in yeast supernatant relative to serum >0.5 with a CV < 30% was used to identify consumed and secreted compounds.

### 2.4 DNA constructs

To express the transporter coding genes in oocytes, genes were cloned downstream of the T7 promoter in the USER compatible *Xenopus* expression vector pUSER016 (Nour-Eldin et al., 2006). The empty vector was digested by PacI and *Nt.BbvCI* (New England Biolabs). The transporter genes were amplified from genomic DNA of CEN.PK113-7D or BY4741, where indicated. The amplified DNA fragments were gel purified and together with the linearized plasmids incubated with USER enzyme (New England Biolabs) for 25 min at 37°C, followed by incubation at 25°C for 25 min. The reactions were transformed into chemically competent *E. coli* cells. All the cloned plasmids were verified by Sanger sequencing. See Supplementary Tables S1–S3 for the sequences of primers and a list of the plasmids constructed in this study (Supplementary Table S4).

### 2.5 Oocyte uptake assay

The selected transporters were cloned into expression vectors using USER cloning. The expression vector contained the T7 transcription initiation site and a poly-A tail and terminator. The full construct was amplified by PCR, and *in vitro* transcribed using the mMessage mMachine T7 transcription kit from Thermo Fisher Scientific. The quality of the generated RNA was analyzed using an Agilent 2,100 Bioanalyzer. Defollicated stage V-IV oocytes from *Xenopus laevis* were purchased from Ecocyte Bioscience (Dortmund, Germany). Individual oocytes were distributed into a 96 well plate with V-bottom containing Kulori buffer pH 7.4, and injected with 50 nL of a mix of 100 ng/μL RNA of GFP and 400 ng/μL RNA of a transporter of interest using a Roboinject 96 well multichannel injection system (Multi Channel Systems MCS GmbH, Reutlingen, Germany). Control oocytes were injected with 100 ng/μL RNA of GFP only. The oocytes incubated for 3 days at 18 °C.

The uptake assay was carried out as described in Nour-Eldin et al. (2006) and Wulff et al. (2019). After preincubation the oocytes were transferred to the assay solution containing Kulori and the compounds at a concentration of 2 mM each, with the pH of the solution adjusted to pH 5. The oocytes were incubated in the assay solution for 3 h at 18 °C. After washing with Kulori pH 7.4, pools of 10 oocytes were transferred to an Eppendorf tube, and the oocytes were disrupted by pipetting together with 50% ice cold MeOH. The oocyte mix was stored for min 2 h at −20°C, and the supernatant collected by centrifugation at 20,000 *g* for 15 min. The supernatant was further diluted with MilliQ to a final concentration of 29% MeOH before the samples were analyzed by LC–MS. LC–MS data was collected on EVOQ EliteTriple Quadrupole Mass Spectrometer system coupled with an Advance UHPLC pump (Bruker, Fremont, CA, United States). See Supplementary Table S5 for further information on the LC–MS method.

### 2.6 Detection of compounds

LC–MS data was collected on EVOQ EliteTriple Quadrupole Mass Spectrometer system coupled with an Advance UHPLC pump (Bruker, Fremont, CA, United States). Samples were kept cold in the CTC HTS PAL autosampler at a temperature of 5.0°C during the analysis. Injection volume was 1 μL, injected onto a Waters ACQUITY HSS T3 C18 UHPLC column (1.8 μm, 2.1 mm x 100 mm long). The column was held at a temperature of 35.0°C. The solvent system used was Solvent A “MilliQ water with 0.1% formic acid” and Solvent B “Acetonitrile with 0.1% formic acid.” The flow rate was 0.400 mL/min with an initial solvent composition of % *A* = 100, % *B* = 0 held until 0.50 min, the solvent composition was then changed following a linear gradient until it reached % *A* = 5.0 and % *B* = 95.0 at 1.00 min. This was held until 2.79 min when the solvent was returned to the initial conditions and the column was re-equilibrated until 4.00 min. The column eluent flowed directly into the Heated ESI probe of the MS which was held at 200°C and a voltage of 4,500 V. MRM Data was collected in positive ion mode. Other MS settings were as follows, Sheath Gas Flow Rate of 50 units, Nebulizer Gas Flow Rate of 50 units, Cone Gas Flow Rate of 20 units Cone Temp was 350°C, and collision gas pressure 1 mTorr.

### 2.7 Data analysis and bioinformatics

The exometabolome data was visualized using Umap, standard settings. We decided to use UMAP as it is often favored over PCA due to its ability to capture non-linear relationships, preserve both local and global structure in the data, produce more distinct clusters, and better separate different groups within the data. Significance was determined with Student’s *t*-test. *P*-levels >0.005 determines significance. Outliers were identified by the interquartile method. After the outliers were identified, they were removed and the statistical analysis redone. Blast was carried out using NCBI protein blast with standard settings. For blast searches in TCDB, Blosum62 was used as scoring matrix, and cut-off values were the standard settings. Data were visualized using UMAP, and analyzed using R and JMP. Phylogenetic tree was calculated using clustal Omega (Sievers et al., 2011) and visualized using iTOL (Letunic and Bork, 2007, 2021).

## 3 Results

### 3.1 Dedicated library of potential yeast xenobiotics transporters

For expression, we selected transporter proteins from secondary active transporter superfamilies known to transport drugs and natural compounds. Bacterial transporter proteins of the drug/metabolite transporter (DMT) superfamily transport natural products and toxins, like camphor, chloroquine, and ethidium bromide (Berninsone and Hirschberg, 2000; Caffaro and Hirschberg, 2006; Västermark et al., 2011). While yeast transporters of the DMT superfamily are largely uncharacterized, we hypothesized that they could include transporters of xenobiotic compounds. The multidrug/oligosaccharide/polysaccharide (MOP) superfamily includes the multi-antimicrobial extrusion (MATE) family, which consists of well-characterized xenobiotic efflux pumps across several organisms (Hvorup et al., 2003; Tanihara et al., 2007; Staud et al., 2013). At the same time, the two MATE transporters present in the yeast genome, YDR338C and Erc1 (Shiomi et al., 1995), have not previously been characterized for the uptake of non-toxic xenobiotic compounds. We also included transporters from the major facilitator superfamily (MFS), specifically the aromatic acid exporter (ArAE) family, and the yeast Equilibrative Nucleoside Transporters (ENT) family. We further reduced the list of transporters by omitting sugar transporters and other experimentally validated nutrient transporters. We also excluded the ABC transporters, as they tend to be exporters rather than importers, and the direction of transport is normally fixed via the internal hydrolysis of ATP.

**Figure 1:**
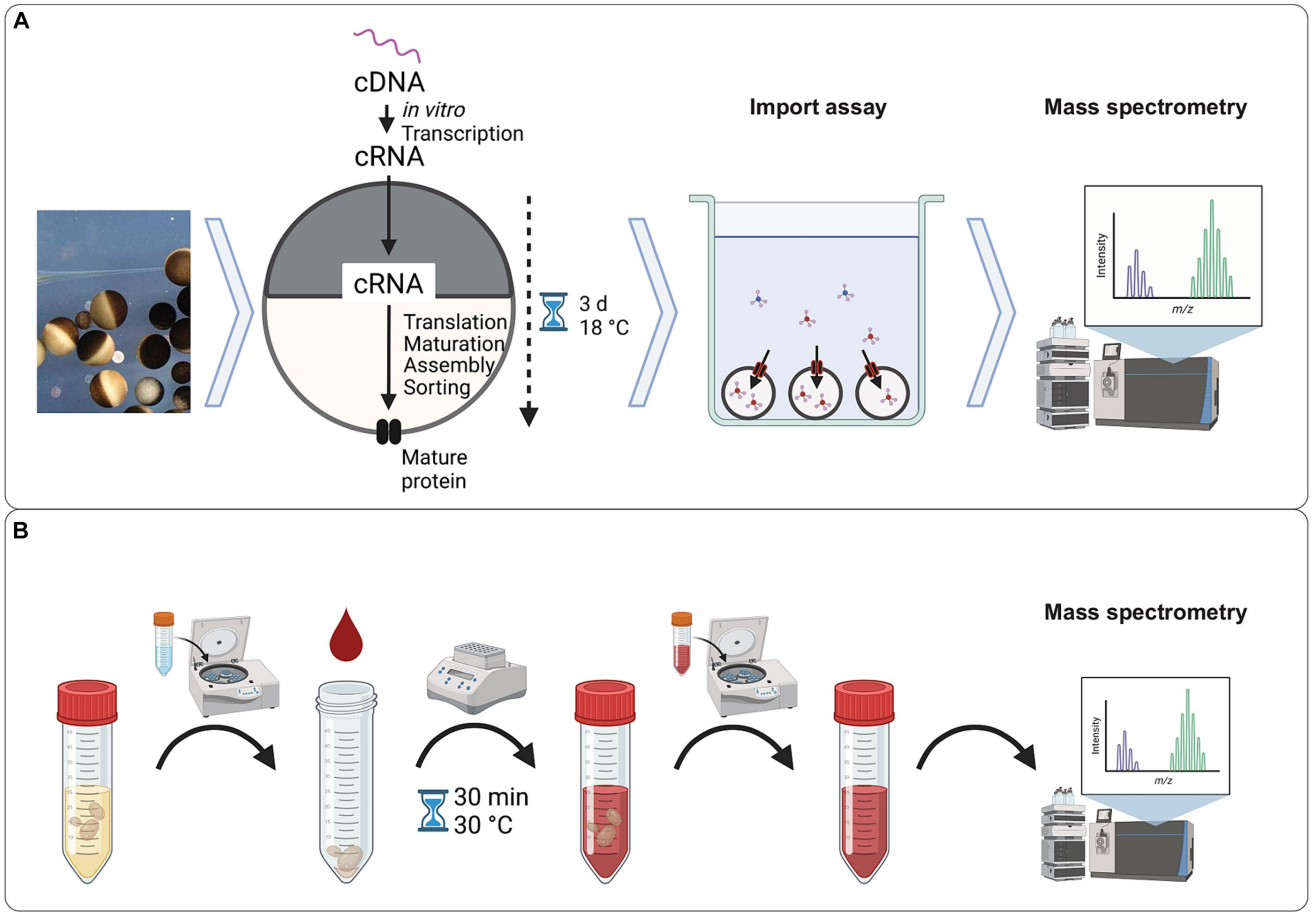
Experimental workflows. **(A)** Workflow of the uptake assay using *Xenopus* oocytes. Defolicated oocytes were injected with mRNA encoding the transporter of interest and mRNA encoding GFP. After 3 days of incubation at 18 °C, the fluorescence from the GFP was quantified, and oocytes with an active expression of GFP were selected for the uptake assay. Oocytes expressing the transporter of interest were exposed to a solution of the selected compounds in Kulori buffer at pH 5.0 for 3 h. After incubation, the oocytes were harvested, and the intracellular content was extracted and analyzed by LC–MS. **(B)** Workflow of the exometabolome assay. An overnight yeast culture was harvested by centrifugation, washed, and resuspended in human blood serum. After 30 min incubation with the human blood serum, the supernatant was harvested and subjected to analysis by LC–MS. Figure generated using BioRender.

The workflow of *Xenopus* oocyte uptake assay is described in Figure 1A. Briefly, the oocytes were injected with mRNA encoding the transporter of interest and mRNA encoding GFP and incubated for 3 days to allow protein expression. Fluorescence was used as a quality control to select for the oocytes that received mRNA and expressed the proteins, supposedly both GFP and transporter. The transporter expressing oocytes were incubated with the solution of several selected xenobiotic metabolites in pH 5 buffer. The low pH was chosen because the yeast *S. cerevisiae* is known to proliferate in an acidic environment. After incubation, the concentration of xenobiotic metabolites was determined in the buffer and in the oocytes by LC–MS.

### 3.2 Compounds transported by the yeast *Saccharomyces cerevisiae*

**Figure 2:**
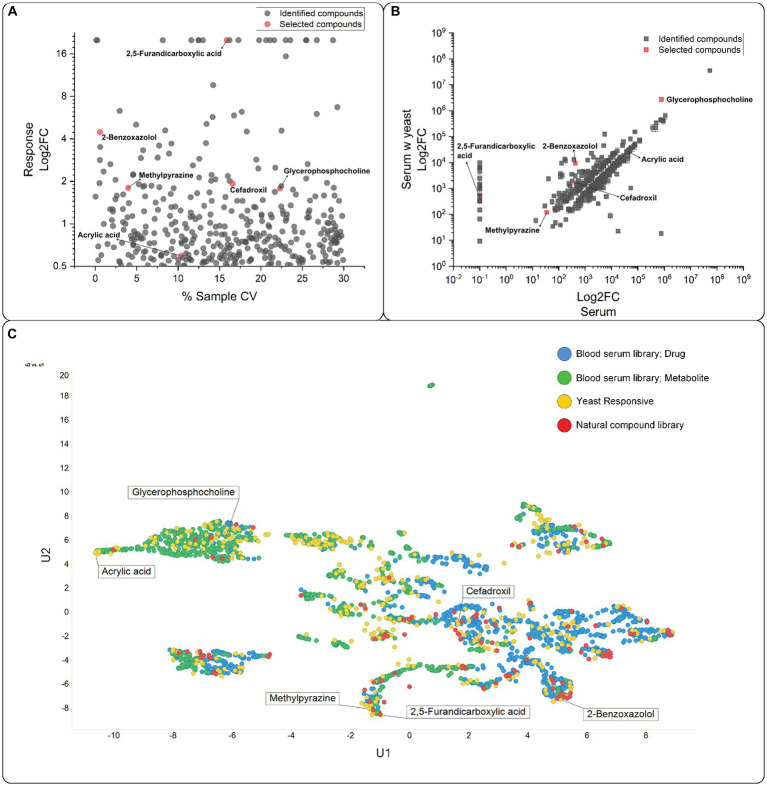
Compounds transported by yeast. **(A)** Variation observed between the repeats. CV, coefficient of variation. Compounds with a response below a log_2_FC = 0.5 were excluded. The selected compounds are highlighted in red. **(B)** The compounds identified in human blood serum were plotted against the compounds identified in the supernatant incubated with yeast. Compounds with a log_2_FC below 0.5 were excluded. Selected compounds are highlighted in red. **(C)** UMAP (McInnes et al., 2018a, 2018b) plot of the compounds with a log_2_FC above 0.5. Selected compounds are indicated with names. The compounds are grouped according to their classification in DrugBank 3.0 (https://www.drugbank.ca) and Recon 2 (Thiele et al., 2013) as detailed in O’Hagan and Kell (2017).

An initial screen to establish the small molecules that yeast cells can transport was conducted (Figure 1B). Yeast cells were incubated in human blood serum for 30 min, removed by centrifugation, and the supernatant analyzed on LC–MS. There was a concentration change (log_2_FC > 0.5) in 385 compounds (Supplementary Dataset S1 and Figure 2A). We observed a considerable variation in response across replicates in the yeast-treated samples compared with the untreated samples (Figure 2A), though not to the extent where the compound changed the direction of transport. The log_2_FC values in concentration compared to the untreated samples were predominantly negative (total of 221 compounds, 169 metabolites and 52 drugs/xenobiotics), indicating the import of compounds from the serum or the metabolism and degradation of compounds. On the other hand, the log_2_FC values were also positive for 164 compounds (97 metabolites and 67 drugs/xenobiotics), suggesting an increase in extracellular concentration due to the export or antiport of yeast metabolites or enzymatic activity on serum compounds (Figure 2B). The UMAP visualization showed a skewness, with compounds categorized as drugs present on the right-hand side of the U1 axis and compounds categorized as metabolites on the left (Figure 2C). This distinction in compound class has seemingly no influence on which compounds yeast interacts with, indicating that in our experiment, yeast had no substrate preference to drugs or metabolites.

Subsequently, we overlaid the identified compounds with a log_2_FC > 0.5 from the exometabolome experiment data with a small natural compound library (O'Hagan and Kell, 2019) (Figure 2C). The overlay revealed that the small compounds categorized as drugs tend to have higher similarity to natural products, which was also previously described (O’Hagan and Kell, 2017), with most marketed drugs and natural products displaying a Tanimoto similarity of 0.7 or higher.

### 3.3 Selected compounds for characterization in transport assays in *Xenopus laevis* oocytes

The 385 compounds were evaluated for their commercial availability, solubility in water, and general commercial interest (see Supplementary Dataset S2). We then tested the top-ranking compounds for toxic effects on the oocytes and excluded compounds that resulted in an above-average death rate. Additionally, compounds not precisely detected by our MS method were also excluded. The six compounds selected for testing were sn-glycero-3-phosphocholine (GPC), 2,5-furandicarboxylic acid (FDC), 2-methylpyrazine (MPZ), cefadroxil (CFX), acrylic acid (ACA), and 2-benzoxazolol (BXZ). The compounds represent metabolites (GPC), compounds with natural origin (MPZ, BXZ, and FDC), and synthetic compounds (CFX and ACA). In the UMAP visualization, the six compounds are evenly distributed (Figure 2C), with MPZ and FDC being the most closely related compounds.

Both FDC and ACA are industrially relevant. FDC is one of the US Department of Energy’s top 12 compounds for establishing a biology-based chemical industry (Bozell and Petersen, 2010), while ACA is an essential monomer in the chemical industry. ACA is approved as a food contact material and acidity regulator (Silano et al., 2018). Both ACA and FDC are formed by excessive heating of food and enter the body via the diet. ACA and its precursor 3-hydropropionic acid have been produced in cell factories (Dishisha et al., 2015). CFX is a broad-spectrum antibiotic with several known transporters in humans [SLC15A1 (Ganapathy et al., 1995; Wenzel et al., 1996), SLC15A2 (Ganapathy et al., 1995; Terada et al., 1997; Ocheltree et al., 2004; Luckner and Brandsch, 2005), SLC22A5 (Ganapathy et al., 2000), SLC22A6 (Jariyawat, 2000; Jung et al., 2002; Takeda et al., 2002), SLC22A8 (Jung et al., 2002; Takeda et al., 2002)]. MPZ is a naturally occurring flavoring agent found in many foods, while BXZ is a compound naturally occurring in cereal and cereal products and rye seedlings. Lastly, GPC is a native metabolite in humans, yeast, plants, and *Xenopus laevis*.

### 3.4 Substrate identification

The six selected compounds were used to screen the transport activity of transporters expressed in *Xenopus* oocytes. For normalization, we used the intracellular concentration of compounds in control oocytes that expressed only GFP, but no yeast transporters.

**Figure 3:**
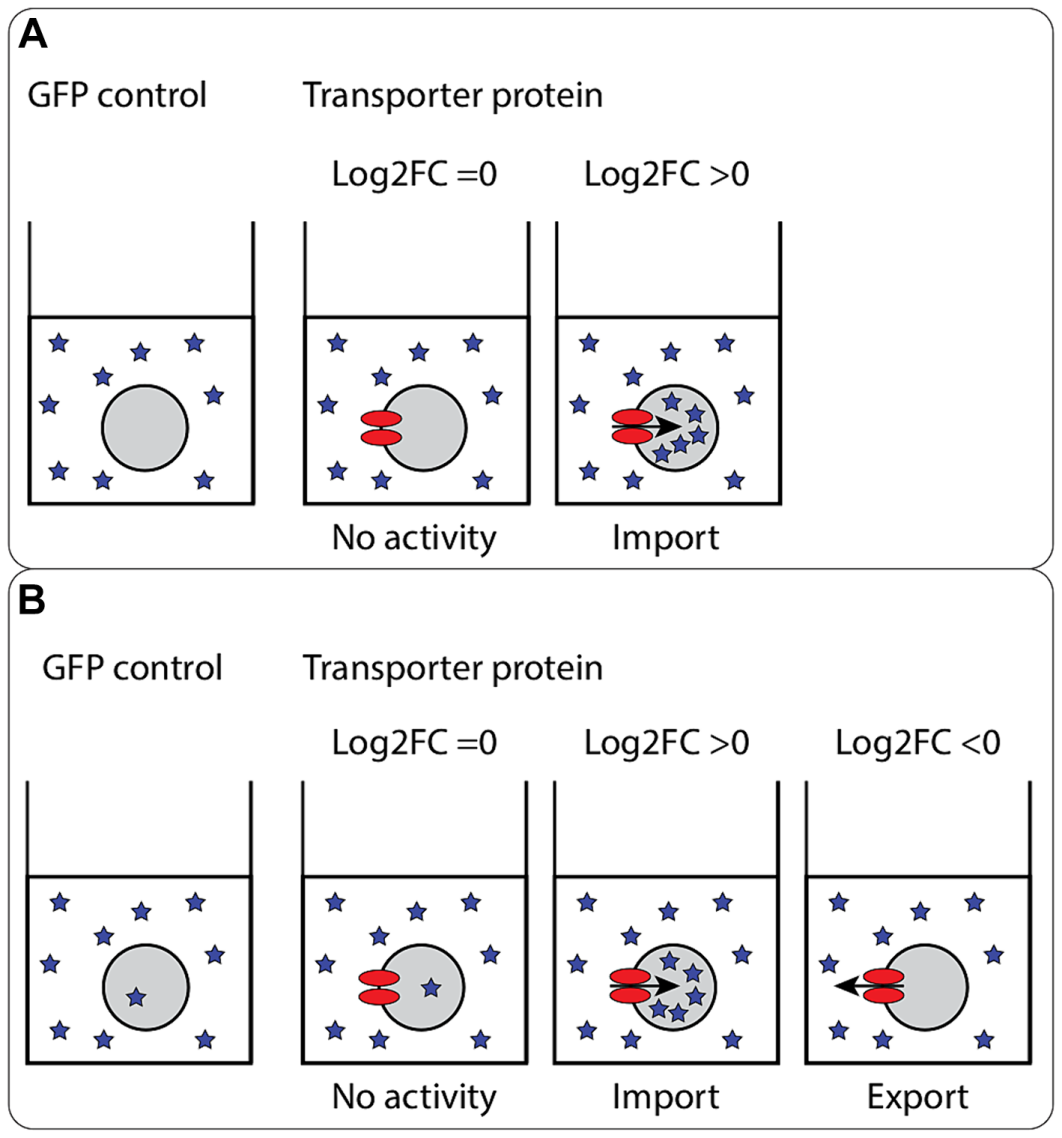
Principles of the oocyte import assay. Schematic representation of the oocyte import assay. For uptake, the intracellular concentration per oocyte is compared. **(A)** No or very little of the compounds are measured in the control oocytes. As no endogenous import activity is detected, only no activity (Log_2_FC = 0) or import activity (Log_2_FC > 0) can be detected for the expressed transporter proteins. The import can be categorized in two groups; concentrative import, where the intracellular concentration reached is higher than the concentration in the medium, and equilibrative import, where the intracellular concentration reached is similar or lower than the concentration found in the medium. **(B)** Some endogenous import activity is detected. With some endogenous import activity, export activity of the expressed transporter proteins can be measured (Log_2_FC < 0), in addition to no activity and import activity. In this scenario, two levels of import can also be reached; concentrative import and equilibrative import.

A positive Log_2_FC is a measure of uptake activity compared to the control. Conversely, a negative Log_2_FC is a measure of export activity compared to the control (Figure 3A). The compounds were added in excess (2 mM), to ensure saturation for the transport protein was reached. Likewise, the incubation time (3 h) was chosen to ensure saturation. Export activity of transporters can be evaluated if the compound is imported into the GFP control by oocyte endogenous transporters or if the compound compromises the membrane of the oocytes and leaks in Figure 3B. Otherwise, export activity measurement would require injection of metabolites into oocytes, so called “export assay,” which was not performed in this study.

**Figure 4:**
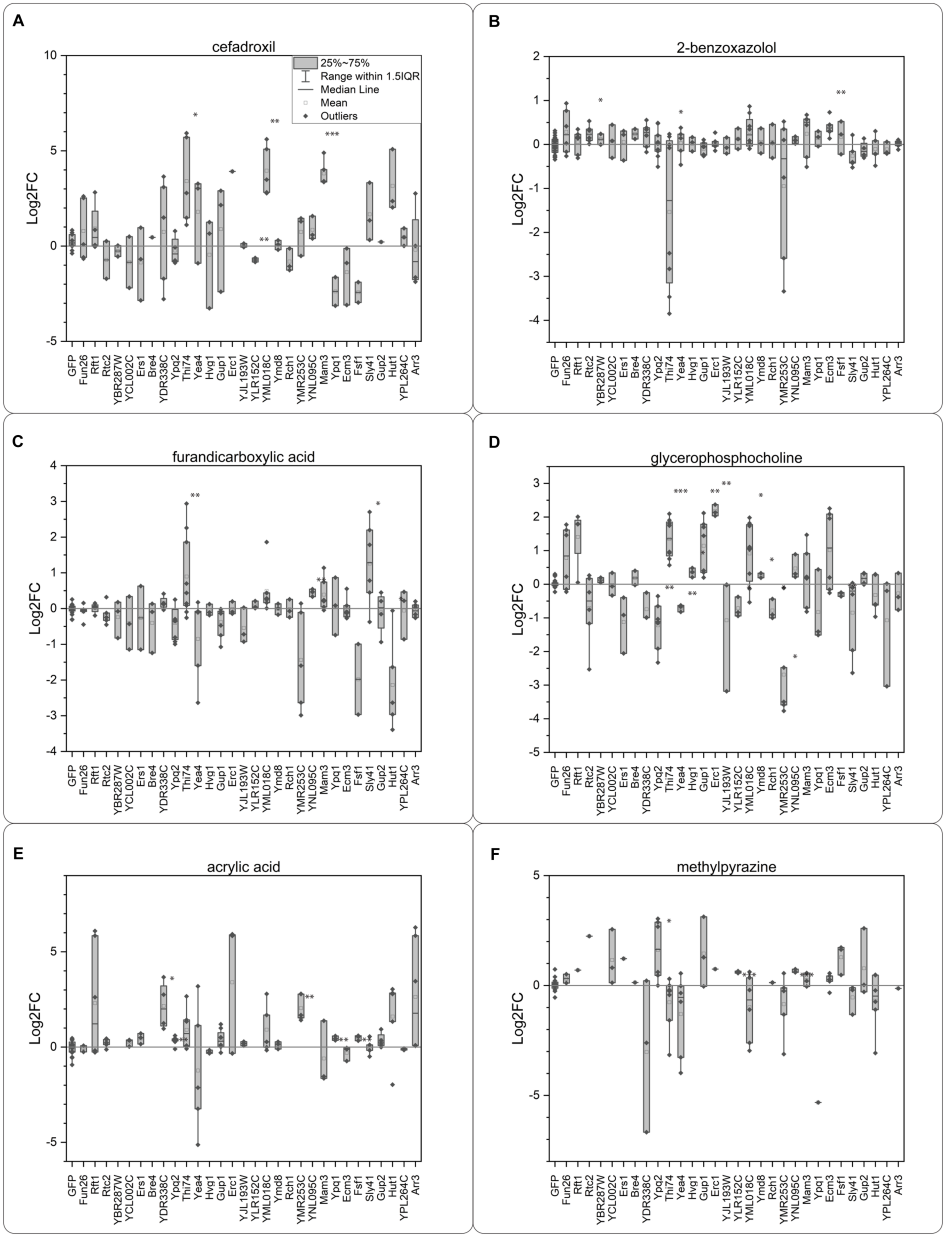
Oocyte import assay. Each compound was added at an extracellular concentration of 2 mM at pH 5.0. **(A)** Cefadroxil, **(B)** 2-benzoxazolol, **(C)** 2,5-furandicarboxylic acid, **(D)** sn-glycero-3-phosphocholine, **(E)** acrylic acid, **(F)** 2-methylpyrazine. The response was calculated as fold change compared to the response of the GFP injected control oocytes. Each data point consists of data from 7 to 10 oocytes in triplicates and each experiment was repeated on several occasions. The intracellular concentration in the single oocytes was calculated depending on the number of oocytes used in the individual experiment and used for calculating the Log2 fold change compared to the GFP control. Each point is a replicate based on extraction data from 10 oocytes. Significance was tested with a two-sided Students *t*-test, for transporters having an intracellular concentration higher than the GFP expressing controls. Significance levels indicated in the graphs are: ^*^p ≤ 0.05, ^**^p ≤ 0.01, ^***^p ≤ 0.001.

We identified transporter candidates with a significant response compared to the GFP control for all the tested compounds (Figure 4). Eight transporter candidates were identified for the three natural compounds, BXZ, MPZ, and FDC. For BXZ, the transporter proteins Rtc2, Thi74, and Ecm3 generated a significant response (Figure 4B). For MPZ, the transporter proteins Ypq2, YLR152C, and YNL095C generated a significant response (Figure 4F), and for FDC, the transporter proteins Thi74, YNL095C, Sly41, and Hut1 generated a significant response (Figure 4C). It is worth noting that Thi74 showed a negative Log_2_FC for BXZ, and Hut1 for FDC, suggesting Thi74 and Hut1 are exporters. All other transporters showed a positive Log_2_FC in intracellular concentration, suggesting the transporter proteins are importers. As prolonged incubation with BXZ and FDC did not result in increased mortality in oocytes, we do not have a reason to believe that BXZ or FDC compromised the oocyte membrane. The considerable variation between samples for Thi74 across the tested substrates could suggest difficulties with expressing Thi74 in oocytes.

For the synthetic compounds ACA and CFX, we identified nine transporters. The transporter proteins YDR338C, Ypq2, YMR253C, Ypq1, and Fsf1, showed a significant response for ACA (Figure 4E). For CFX, we identified both proteins in the paralog pair Thi74 and YML018C as candidates, together with the transporter proteins Mam3 and YLR152C (Figure 4A).

While all the compounds mentioned were identified as substrates for between 2 and 4 transporters, the metabolite GPC was the substrate of 12 transporters (Figure 4D). We identified both paralogs Thi74 and YML018C as GPC transporter candidates, but only Gup1 from the paralog pair Gup1/Gup2. Other identified GPC candidates were Ypq2, Yea4, Hvg1, Erc1, YLR152C, Ymd8, Rch1, YMR253C, and Fsf1. Ypq2, Yea4, YLR152C, and Fsf1 all showed a negative Log_2_FC in intracellular concentration, suggesting the transporter proteins are exporters.

### 3.5 Transporter proteins allow the passage of xenobiotics in both a concentrative and equilibrative manner

**Figure 5:**
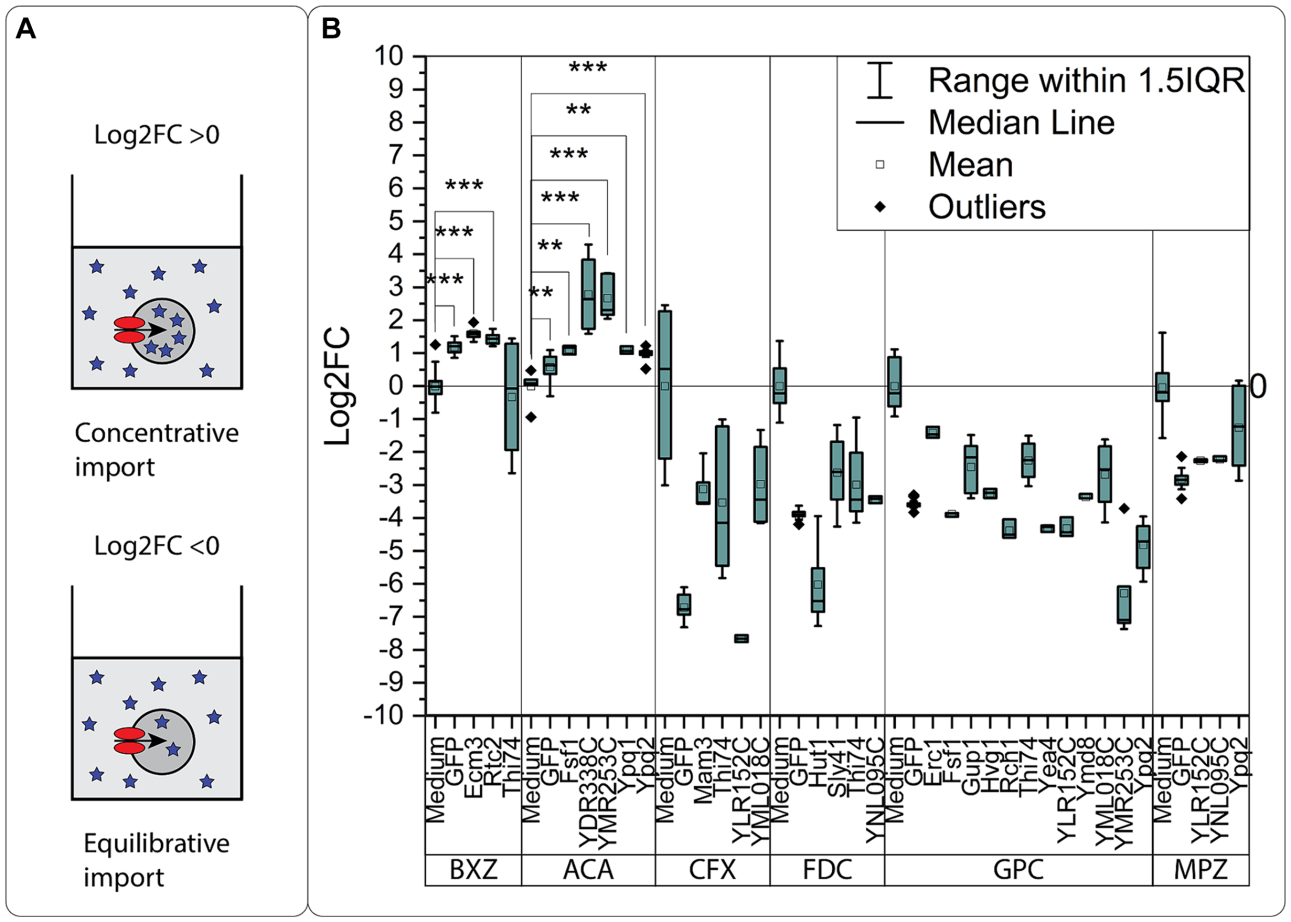
The mode of transport. **(A)** Schematic representation of the comparison with the medium concentration. A Log_2_FC > 0 indicates a concentrative mode of transport, as the internal concentration in the oocytes exceeds the concentration found in the medium. A Log_2_FC < 0 indicates an equilibrative mode of transport, as the internal concentration found in the oocyte does not exceed the concentration found in the medium. **(B)** The significant transporters identified in the oocyte uptake assay are plotted together with the relative concentration found in the medium. All values are calculated as Log_2_FC compared to the extracellular concentration found in the medium. Significance was tested with a one-sided Students *t*-test, for transporters having an intracellular concentration higher than the medium. Each point is a replicate based on extraction data from 10 oocytes. Significance levels indicated in the graphs are: ^*^p ≤ 0.05, ^**^p ≤ 0.01, ^***^p ≤ 0.001.

To describe the thermodynamic nature of the transport mechanism of the identified transporter proteins acting as importers [i.e., equilibrative vs. concentrative (Kell and Oliver, 2014)], we compared the normalized intracellular concentration of the compounds found in the oocyte with the extracellular concentration found in the medium (Figure 5A). As the compounds were added in excess, and the volume of the medium was significantly higher than the volume of the oocytes, we assumed that the concentration found in the medium was constant for all experiments. A Log_2_FC > 0 suggests a concentrative mode of transport, indicating that the internal concentration within the oocytes surpasses that found in the medium. Conversely, a Log_2_FC < 0 indicates an equilibrative mode of transport, where the internal concentration within the oocyte does not surpass that found in the medium. The comparison revealed that only the transporters of BXZ (Ecm3, Rtc2) and ACA (Fsf1, YDR338C, YMR253C, Ypq1, and Ypq2) import their substrates in a concentrative manner (Log_2_FC > 0) (Figure 5B), while CFX, FDC, GPC, and MPZ as substrates are effectively imported in an equilibrative manner (Log_2_FC < 0) (Supplementary Table S5). However, when the GFP expressing control is taken into consideration, it becomes apparent that the transporters of BXZ and ACA have a systemic bias. For BXZ and ACA, the GFP control has a Log_2_FC > 0. This indicates uptake of the compounds by an endogenous transporter. While subtracting the effect from endogenous transporters suggests YDR338C and YMR253C transport ACA concentratively, further experiments are needed to verify the results.

In the oocyte uptake assay, we identified GPC as the substrate for the highest number of transporters (12). The comparison with the concentration found in the medium revealed that all transporters transported GPC equilibratively. We previously observed that GPC could enter the oocyte through an unknown mechanism, possibly through leakage or through an endogenous transporter. We rule out the possibility that the oocytes are compromised in general by GPC, as we did not detect all six compounds intracellularly in the GFP-expressing control oocytes.

If GPC is excluded, all concentrative transporters but Ypq2 transported only one compound (ACA), strictly in a concentrative manner. The remaining equilibrative transporters transported from one substrate (Sly41/FDC) up to three substrates for Thi74 (BXZ, CFX, FDC). The concentrative nature of the ACA transporters would suggest they are either primary or secondary active transporters, able kinetically and thermodynamically to transport their substrate in a uni-directional way under the conditions tested.

Examining the direction of transport found in the data from the exometabolite assay, ACA was found to be effectively imported rather than exported. Our results suggest that ACA, in general, is imported into the cell by the transporters YMR253C, YDR338C, Fsf1, Ypq2, and Ypq1, where YMR253C and YDR338C potentially transport ACA in a concentrative manner. When further assessing the oocyte uptake data, we identified Thi74 as an exporter of BXZ rather than an importer. In the exometabolite assay, we had identified BXZ to be effectively exported, meaning either Ecm3 and/or Rtc2 transports BXZ in a bi-directional manner, or other transporters exist, including Thi74. For CFX, FDC, and MPZ, all three compounds were found to be effectively exported in the exometabolome assay. In the oocyte uptake assay, we had a higher extracellular concentration, identifying solely equilibrative transport for CFX, FDC and MPZ.

Overall, we found the equilibrative transport of compounds to be a widespread phenomenon among the tested transporters and more common than concentrative transport. Moreover, we found that the ability to transport a compound concentratively was not restricted to a specific compound class, though assessing a larger chemical library is needed to investigate the phenomenon further.

### 3.6 Effect of transporter deletions in *Saccharomyces cerevisiae*

**Figure 6:**
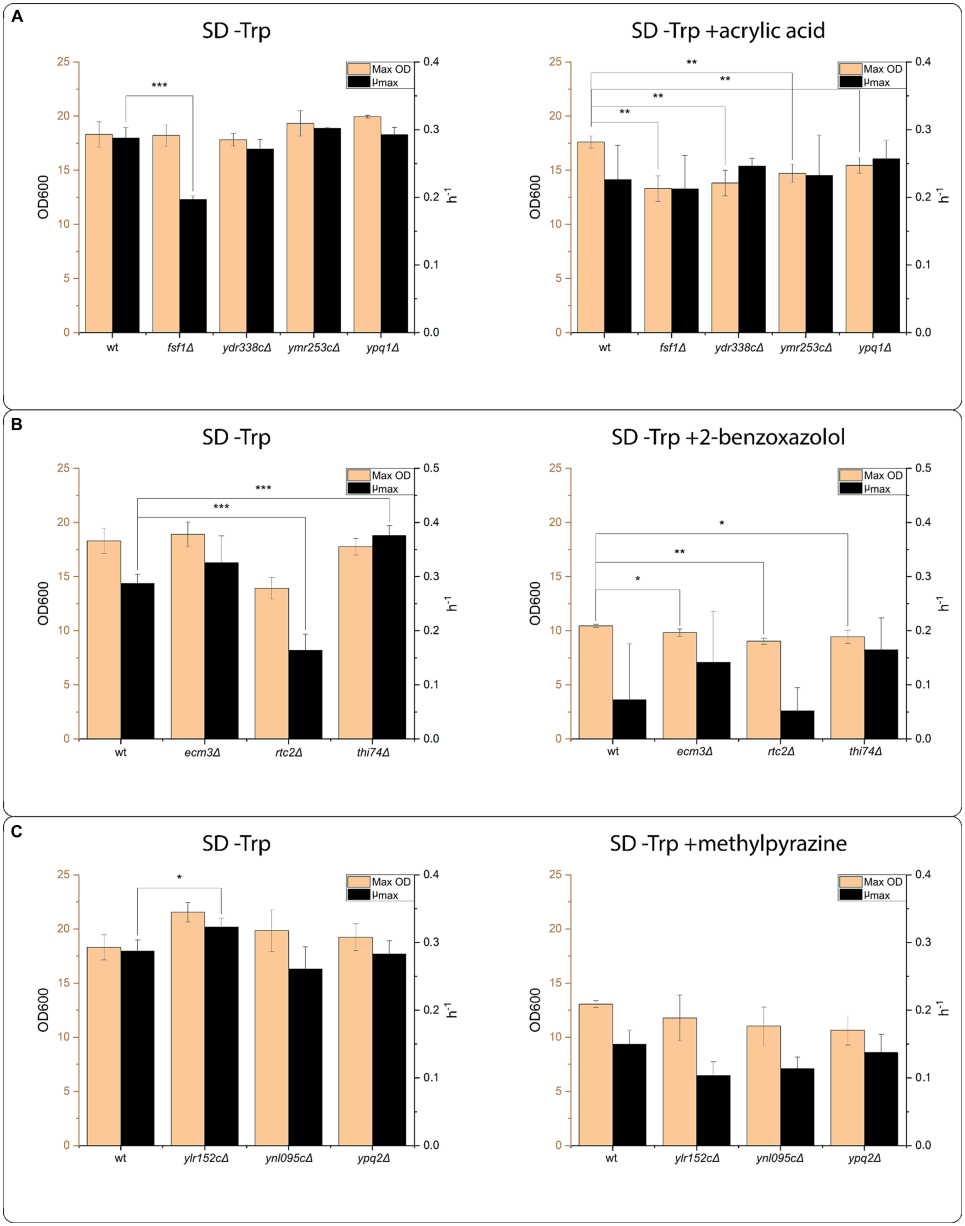
Transporter mutant assays. Growth assay of strains with the transporter proteins identified in the screening effort. From the growth curves generated in the growth profiler, maximum OD reached (Max OD), and maximum specific growth rate (μ_max_) were calculated. All strains were grown in the medium composition SD –Trp, and in SD –Trp supplemented with the indicated compounds at LC50 concentration. The LC50 concentration was established in a pre-experiment using the wildtype BY4741 strain. **(A)** Growth assay of strains harboring deletions of transporters genes identified in the screening effort as transporter proteins having transport activity toward acrylic acid. The assay was conducted with 2.45 mM acrylic acid. **(B)** Growth assay of strains harboring deletions of transporters genes identified in the screening effort as transporter proteins having transport activity toward 2-benzoxazolol. The assay was conducted with 6.44 mM 2-benzoxazolol. **(C)** Growth assay of strains harboring deletions of transporters genes identified in the screening effort as transporter proteins having transport activity toward methylpyrazine. The assay was conducted with 155.15 mM methylpyrazine. Significance levels indicated in the graphs are: ^*^p ≤ 0.05, ^**^p ≤ 0.01, ^***^p ≤ 0.001.

We further tested the effect of the identified transporters from the oocyte screen *in vivo* by growing *S. cerevisiae* strains harboring deletions of the single genes in medium with the supplementation of one of the six selected compounds. The strains were grown at the LC50 concentration of the compounds, to allow both improvements in growth and growth inhibitions to be detectible (the data forming the basis for the LC50 calculations is available in Supplementary Table S6). We were not able to obtain a growth defect approximating to 50% for the reference BY4741 strain grown in SD-Trp supplemented with GPC or CFX. Therefore, we omitted the two compounds from the final growth experiment. We also omitted FDC from the final growth experiment, as the compounds precipitated at the calculated LC50, which biased the results. Using the calculated LC50 concentrations for ACA, BXZ and MPZ, strains harboring deletions of the identified transporters were cultivated in SD-Trp with and without the compounds in a growth profiler, the growth curves can be found in Supplementary Figures S1–S4. Figure 6 shows the calculated maximum OD (Max OD) and the maximum specific growth rate (μ_max_) using the growth curves For acrylic acid, the strain harboring a deletion of FSF1 (*fsf1∆*), showed a decrease in μ_max_ rate when grown in SD-Trp (Figure 6A). The decrease in growth rate was rescued in the presence of acrylic acid. In addition, there was no difference in maximum OD for the ACA transporters deletion strains in SD-Trp, while when grown in the presence of ACA, all the ACA transporters deletion strains had a significantly lower maximum OD compared to the wildtype strain (Figure 6A).

For BXZ transporters, *rtc2∆* mutant had lower μ_max_ and *thi74∆* had higher μ_max_ in SD-Trp than the reference strain (Figure 6B). In the presence of BXZ, neither of the three strains tested had a significant difference in μ_max_, while all tested strains had a significant decrease in maximum OD. For MPZ transporters, only YLR152C∆ exhibited a growth phenotype (Figure 6C). YLR152C∆ showed a significant increase in μ_max_ compared to the reference when grown on SD-Trp, while this μ_max_ was not significantly different from the reference when grown in the presence of MPZ (Figure 6C).

### 3.7 Sequence-function relationship

For transporters, divergent evolution is overwhelmingly the most common means of evolution, i.e., similar sequences evolve to transport different substrates (Saier, 1994; Wang et al., 2016; Jørgensen et al., 2017). To look into the conservation of substrate specificity among the identified transporters, we generated a phylogenetic tree as well as assessed their transporter classification for indications of a potential co-substrate.

**Figure 7:**
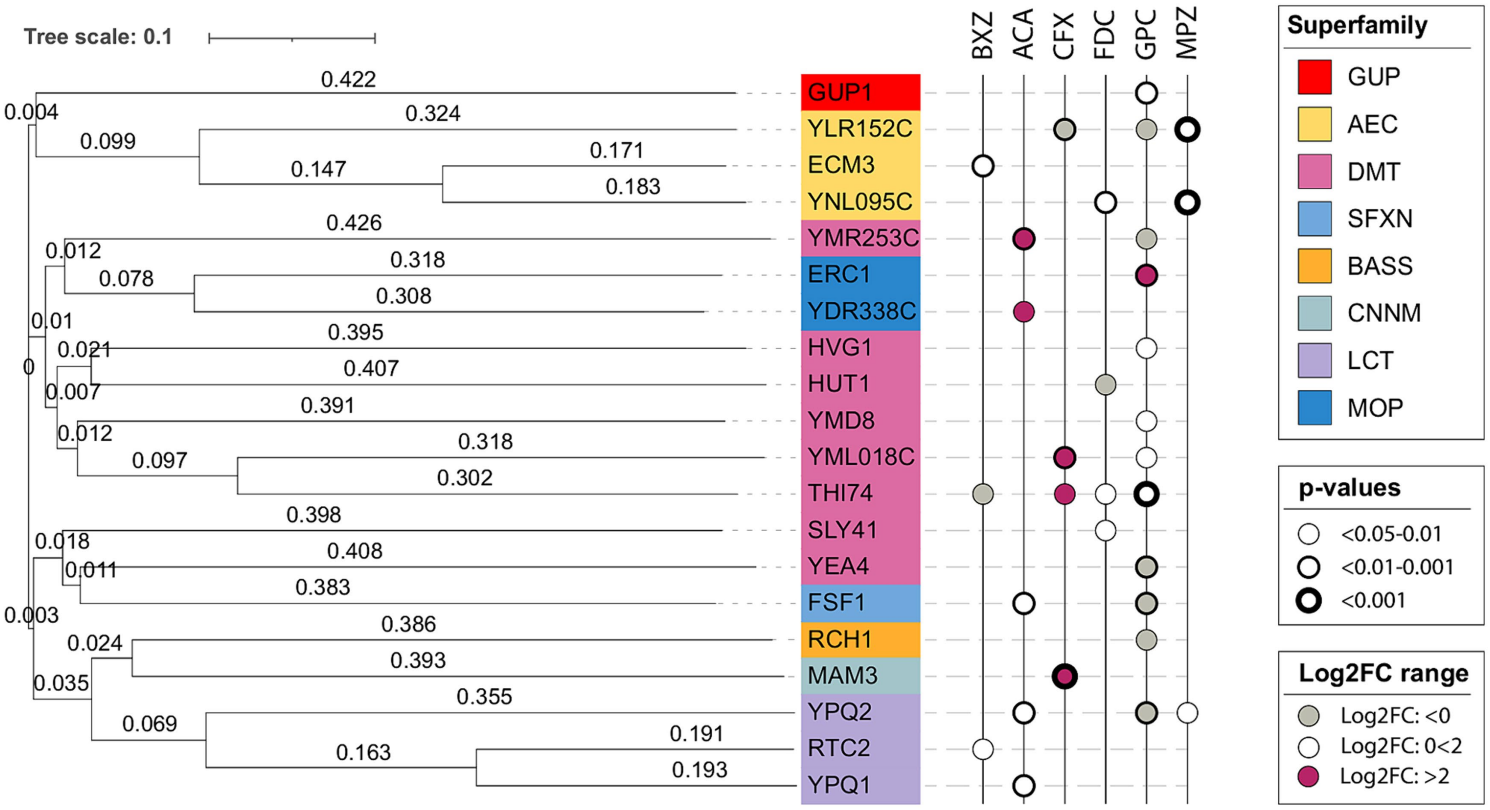
Phylogenetic tree of tested transporters with identified substrates. The tree was generated with Clustal Omega (Sievers et al., 2011) version 1.2.4 using the standard parameters. The generated phylogenetic tree was visualized with iTOL (Letunic and Bork, 2007, 2021). The transporter proteins are color-coded according to superfamily. The transporter superfamilies are abbreviated as follows: the glycerol uptake (GUP) superfamily, the auxin efflux carrier (AEC) family from the bile/arsenite/riboflavin transporter (BART) superfamily, drug/metabolite transporter (DMT) superfamily, the sideroflexin (SFXN) family, the bile acid: Na^+^ symporter (BASS) family, the cyclin M Mg^2+^ exporter (CNNM) family, the lysosomal cystine transporter (LCT) family, the multidrug/oligosaccharidyl-lipid/polysaccharide (MOP) flippase superfamily.

Among the chosen transporters were a few pairs of paralogs with a considerable sequence similarity: Thi74/YML018C, Rtc2/Ypq1, Gup1/Gup2, Ecm3/YNL095C (Figure 7). For Thi74 and YML018C, we identified CFX and GPC as shared substrates, while FDC and BXZ were unique to Thi74. Both Rtc2/Ypq1 and Gup1/Gup2 failed to show an overlap in the substrate. The lack of functional overlap for the paralog pairs could suggest evolutional diversification through relatively few mutations. Indeed, paralog transporters have been previously shown to have different substrate specificities (Ramaswamy et al., 2017; Jiang et al., 2023). Consequently, the substrate of a transporter protein is hard to predict based on the amino acid sequence alone. There was no clear correlation between sequence similarity and substrate specificity in our study.

As a means of further validating our results, we sought to verify the presence of co-substrates for the transporter families. The presence of a co-substrate would indicate whether there is a known source of electrochemical power and hence would suggest the transporter can transport substrates concentratively. Assessing the identified transporters’ TCDB classification (Saier, 2006; Saier et al., 2016) did not give any further insight. All transporters, except Mam3, belong to the pores transporter class, including both symporters, uniporters, and antiporters (Supplementary Table S7), giving no additional information concerning potential co-substrates.

## 4 Discussion

We applied a workflow combining exometabolome analysis and *Xenopus* expression to map substrate-transporter relationships for six metabolites and 30 yeast transporters. The fact that we could identify transporters for all selected compounds was surprising considering that we only tested 30 transporters, which comprises only a tenth of all the yeast transporters. Of the 20 transporter proteins identified in this screen, Sly41, YML018C, Mam3, and Ecm3 have not previously been assigned a substrate experimentally.

In transporter mutant assays in *S. cerevisiae* we observed a growth phenotype for most of the strains, which further strengths the screening approach using *Xenopus* oocytes. We did however not observe a growth phenotype for all the tested transporters. The lack of growth phenotype could be due to the localization of the transporter. If the transporter and the compound are not localized within the same cellular compartment, their interaction (transport) cannot occur, and consequently, observable effects may not manifest. However, it does not exclude the transporter protein from having activity against the compound. The observed growth phenotype of the transporter deletion strains indicates the transporters are involved in resistance toward ACA, BXZ and MPZ (though only Ylr152c) at the tested concentration. The observed alleviation of the slower growth rate for FSF1∆ in the presence of ACA, and RTC2∆ for BXZ could imply a potential association of Fsf1 and Rtc2 with the import or availability of these compounds. Fsf1, identified as a mitochondrial protein (Reinders et al., 2006), may likely contribute to the availability of ACA at its intramitochondrial target, where it may exert its toxic effects, rather than being directly involved in its import into the cell. Similarly, Rtc2, proposed as a probable vacuolar protein (Jézégou et al., 2012), aligns with a potential role in facilitating the availability of BXZ in the cytoplasm rather than its direct import. However, the exact relationship between the function and the subcellular localization of these transporters warrants further investigation for a comprehensive understanding.

The significant decrease in maximum OD when the strains are grown in the presence of the compound is in line with a function in export of the compound, or making the compound unavailable at the site of action. This is the case for Ydr338c, ymr253c and Ypq1, for ACA, and Ecm3, Rtc2 and Thi74 for BXZ. For THI74∆ on BXZ and YLR152C∆ on MPZ, the μ_max_ was significantly higher than the wildtype when grown on SD-Trp, while when grown in the presence of the appropriate compound, the μ_max_ was not significantly different. Thi74 is a mitochondrial carrier (Mojzita and Hohmann, 2006) which corresponds well with a function in making BXZ unavailable at the site of action. While Thi74 exhibited bi-directional transport depending on the substrate in the oocyte experiments, similar activities have been observed in other transporters (Lai, 2013). Ylr152c is a protein of unknown function meaning the observed decrease in μ_max_ in the presence of the compounds could suggest Ylr152c is involved in exporting MPZ, or making MPZ unavailable at the site of action.

While we identified a range of transporter proteins for the tested substrates, we only found six transporter proteins to transport their substrates with a Log_2_FC above two. This suggests there are many solute carriers with the capacity to transport xenobiotics from many different chemical classes when the compounds are present in high concentrations, while the capacity to transport the compounds at an efficiency above Log_2_FC is much rarer. The presence of multiple promiscuous transporter proteins in yeast allows the passage of a diverse range of compounds found in the environment. Thereby the yeast cell can ‘sample’ its environment without energy expenditure in the form of ATP or electrochemical gradients. As the transport is equilibrative, the compounds will not accumulate inside the cell. In case of toxicity, the yeast possesses a variety of ATP-driven ABC transporters for efficient multidrug efflux (Egner and Kuchler, 1996; Ortiz et al., 1997; Prasad and Goffeau, 2012; Liu et al., 2023).

The GPC was the most utilized substrate, with 12 transporter proteins showing activity toward GPC. However, more validation of these results is needed because GPC may have impacted the oocyte membrane or had some other secondary effect. Phospholipids, such as GPC, have been shown to alter the function of specific transporter proteins (Stieger et al., 2021). In this context, alternative *in vitro* assays, particularly those employing liposomes and solid-supported membrane-based electrophysiology, represent invaluable tools for comprehensive transporter characterization (Groeneveld et al., 2010; Bazzone et al., 2016, 2017). These methodologies facilitate in-depth investigations into transport mechanisms, substrate specificity, pH dependency, and other critical parameters. Such systems hold promise for further validating or exploring perplexing findings observed with challenging-to-express transporters in oocytes, such as Thi74, and confirming differential substrate specificities observed in transporter paralogs. Furthermore, they could provide avenues to explore aspects such as the absence of structural similarity between our identified and already known substrates and the lack of clear relationships between transporter families and metabolite structures. The prevalence of promiscuous equilibrative transporters has significant implications for strain development in biotechnology. Transporter engineering has shown notable improvements in yeast cell factories for various compounds such as resveratrol (Pan et al., 2008; Vos et al., 2015), squalene (Liu et al., 2023), muconic acid (Pereira et al., 2019; Wang et al., 2021), and betaxanthin (Wang et al., 2021). Several reviews provide additional insights into this progress [see Kell et al. (2015), Jezierska and Van Bogaert (2017), Kell (2018), van der Hoek and Borodina (2020), and Belew et al. (2022)]. However, in certain cases, transporter engineering did not achieve the desired outcomes. For example, with ergothioneine (van der Hoek et al., 2019, 2022) and lactic acid (Fox et al., 2000; Branduardi et al., 2006; Pacheco et al., 2012; Baek et al., 2016; Baldi et al., 2021). The lack of consistent success with transport engineering could be explained by sufficient non-specific secretion of the products by multiple native transporters through equilibrative mechanism, as the product concentration inside the producing cells would typically be higher than the extracellular concentration. The effective extracellular concentration of the product can also be decreased by precipitation or by biphasic fermentation, thus maintaining the product concentration gradient that promotes secretion even when high amounts of product are present in the medium. Resveratrol and muconic acid have low solubility in water and readily precipitate during the process. Squalene is a water-insoluble terpenoid that requires a biphasic fermentation to maintain high titers (Liu et al., 2023). Notably though, if transport can be changed to happen with higher energetic efficiency, then significant improvements in the cell factories by transporter engineering can be achieved. The production of L-glutamic acid (used as taste enhancer monosodium glutamate) was achieved in the 50s’ by corynebacteria when process conditions were discovered that activated a mechanosensitive channel for glutamate export (Nakamura et al., 2007; Sano, 2009). Another example is the discovery of *Schizosaccharomyces pombe* malic acid transporter MAE1 which significantly improved the secretion of dicarboxylic acids in yeast cell factories (Jansen et al., 2012). It was later found that this transporter was likely a voltage-dependent slow-anion channel operating with high energy efficiency, without the use of proton or Na^+^ motive force (Darbani et al., 2019).

In summary, our study provides insights into high transporter promiscuity when it comes to equilibrative transport. This promiscuity has implications both for drug development and for biotechnology and calls for further studies of the phenomenon.

## Data availability statement

The original contributions presented in the study are included in the article/Supplementary material, further inquiries can be directed to the corresponding author.

## Ethics statement

Ethical approval was not required for the studies on animals in accordance with the local legislation and institutional requirements because only commercially available established cell lines were used.

## Author contributions

IM-H: Writing – original draft, Writing – review & editing, Conceptualization, Formal analysis, Investigation, Visualization. JS-S: Writing – review & editing, Investigation. SH: Investigation, Writing – review & editing. JD: Formal analysis, Writing – review & editing. HC: Formal analysis, Writing – review & editing. MW: Formal analysis, Writing – review & editing. SO’H: Visualization, Writing – review & editing. DK: Conceptualization, Funding acquisition, Supervision, Writing – review & editing. IB: Conceptualization, Funding acquisition, Supervision, Writing – review & editing.
