# Supplementary material for: Deorphanizing solute carriers in Saccharomyces cerevisiae for secondary uptake of xenobiotic compounds
Source: Front Microbiol. 2024 Apr 12;15:1376653. doi: 10.3389/fmicb.2024.1376653 (PMC11045925; doi:10.3389/fmicb.2024.1376653)
Supplement: Supplementary file 1 [file Table_1.DOCX]

Supplementary Material

# Supplementary Data

## Dataset S1

Yeast uptake of metabolites in blood serum

## Dataset S2

Selected compounds

# Supplementary Figures and Tables

## Table S1

Cloning primers forward

| Transporter | BioBrick no | Name Fw primer oocyte | Fw primer sequence |
| --- | --- | --- | --- |
| YAL022C | BB2791 | PR-21941 | GGCTTAAUATGAGTACTAGTGCGGACACTG |
| YBL020W | BB2792 | PR-21942 | GGCTTAAUATGGCGAAAAAAAACTCACAATTG |
| YBR147W | BB2793 | PR-21943 | GGCTTAAUATGAAGCTGATCCCAATTATTTTG |
| YBR235W | BB2794 | PR-21944 | GGCTTAAUATGGTTAGTAGGTTTTATCAG |
| YBR287W | BB2795 | PR-21945 | GGCTTAAUATGGTTGAAACATTTAGTTTTGC |
| YCL002C | BB2796 | PR-21946 | GGCTTAAUATGCTTGTTATTGTTCTGCAGGGC |
| YCR075C | BB2798 | PR-21948 | GGCTTAAUATGGTGTCGTTAGACGATATAC |
| YEL004W | BB2799 | PR-21949 | GGCTTAAUATGTGGAACTCACTAAAAGCATTC |
| YER039C | BB2800 | PR-21950 | GGCTTAAUATGATTTATACGTCGTCAAAGAGC |
| YHR032W | BB2802 | PR-21952 | GGCTTAAUATGTCTAAACAATTTAGTCATACC |
| YDL231C | BB2808 | PR-21958 | GGCTTAAUATGAAAAGATATGAGCGAGATCG |
| YDR338C | BB3126 | PR-21961 | GGCTTAAUATGGCTGGAATTTTGTCAAAGAC |
| YDR352W | BB3127 | PR-21962 | GGCTTAAUATGTCGTGCTCAAACGGCATCTGG |
| YDR438W | BB3129 | PR-21964 | GGCTTAAUATGAATCGTGTTGGTATAGACG |
| YGL084C | BB3130 | PR-21965 | GGCTTAAUATGTCGCTGATCAGCATCCTGTC |
| YGL140C | BB3131 | PR-21966 | GGCTTAAUATGTCGCTTAAATCAAAGTTGAC |
| YJL193W | BB3133 | PR-21968 | GGCTTAAUATGTTTCAACAGCTGTCGGC |
| YLR152C | BB3136 | PR-21971 | GGCTTAAUATGTCCCTTTCTCTGGGTGCCGC |
| YML018C | BB3138 | PR-21973 | GGCTTAAUATGGTGTCGAAGGATCAAACGTCC |
| YML038C | BB3139 | PR-21974 | GGCTTAAUATGAATAGGACTGTCTTTTTGGC |
| YMR034C | BB3140 | PR-21975 | GGCTTAAUATGAAGACTCAGTACTCTCTAATA |
| YMR253C | BB3141 | PR-21976 | GGCTTAAUATGAATCCATCAGTACCGAAGG |
| YNL095C | BB3143 | PR-21978 | GGCTTAAUATGGTGCACATTACTCTGGGTC |
| YOL060C | BB3145 | PR-21980 | GGCTTAAUATGTCGTTTTTGCCACTAAGGTC |
| YOL092W | BB3147 | PR-21982 | GGCTTAAUATGCAACTTGTGCCGCTAG |
| YOR092W | BB3149 | PR-21984 | GGCTTAAUATGACACACATCACACTGGGAC |
| YOR271C | BB3151 | PR-21986 | GGCTTAAUATGGCATCATCAGTCCCAGGGC |
| YOR307C | BB3152 | PR-21987 | GGCTTAAUATGATTCAAACGCAAAGTACAGCG |
| YPL189W | BB3156 | PR-21991 | GGCTTAAUATGTCGATGTTAAGAATCTGGAG |
| YPL244C | BB3157 | PR-21992 | GGCTTAAUATGGCGGGAAGTACATCCAG |
| YPL264C | BB3158 | PR-21993 | GGCTTAAUATGACGCTGCAAAGAATTAGTAAA |
| YPR201W | BB3160 | PR-21995 | GGCTTAAUATGTCAGAAGATCAAAAAAGTG |
| yEGFP | BB2959 | PR-21996 | GGCTTAAUATGTCTAAAGGTGAAGAATTATTC |

## Table S2

| Cloning primers reverseTransporter | BioBrick no | Name Rw primer oocyte | Rv primer sequence |
| --- | --- | --- | --- |
| YAL022C | BB2791 | PR-21997 | GGTTTAAUTCACCTGATAATAAAGTCAATTATG |
| YBL020W | BB2792 | PR-21998 | GGTTTAAUTCATACATCTTTAGAATTGGAAACCG |
| YBR147W | BB2793 | PR-21999 | GGTTTAAUTCAATTATCTATTAGTATTTTCTCG |
| YBR235W | BB2794 | PR-22000 | GGTTTAAUTCATAATGCAGTAGTTACTGTC |
| YBR287W | BB2795 | PR-22001 | GGTTTAAUTCAAGTTGGGTTAGCCCACTG |
| YCL002C | BB2796 | PR-22002 | GGTTTAAUTCATAGTTTTCCTTTTGGCAACCGTGG |
| YCR075C | BB2798 | PR-22004 | GGTTTAAUTCACAGCGGGTACTCTGACGC |
| YEL004W | BB2799 | PR-22005 | GGTTTAAUTCATTTACTTTTCTTTATCGCCCC |
| YER039C | BB2800 | PR-22006 | GGTTTAAUTCACTTCTCAAGAGTAGCTGCTAG |
| YHR032W | BB2802 | PR-22008 | GGTTTAAUTCAGTTATACCCAACCATAAGCC |
| YDL231C | BB2808 | PR-22014 | GGTTTAAUTCAAAAGTTTTCTTCGCCCAGTATATC |
| YDR338C | BB3126 | PR-22017 | GGTTTAAUTCAAGCCCCTAAGAGGGCTGTG |
| YDR352W | BB3127 | PR-22018 | GGTTTAAUTCATGTTTGCGTTTCACCAGACAAC |
| YDR438W | BB3129 | PR-22020 | GGTTTAAUTCAGGCATCCAAGATAGGTCC |
| YGL084C | BB3130 | PR-22021 | GGTTTAAUTCAGCATTTTAGGTAAATTCCGTGC |
| YGL140C | BB3131 | PR-22022 | GGTTTAAUTCAGTCCTTCTTTTCTTTCATGTG |
| YJL193W | BB3133 | PR-22024 | GGTTTAAUTCACTCAGGACGTATTTTCGC |
| YLR152C | BB3136 | PR-22027 | GGTTTAAUTCAAACTTTTAAATCGACCTTTAAGG |
| YML018C | BB3138 | PR-22029 | GGTTTAAUTCAGTTGTTTGCTGCTGGCACCTC |
| YML038C | BB3139 | PR-22030 | GGTTTAAUTCAGACCTGCTGGGATGAACGAC |
| YMR034C | BB3140 | PR-22031 | GGTTTAAUTCACCTTGGTTGTGTATATGGGAC |
| YMR253C | BB3141 | PR-22032 | GGTTTAAUTCATTTAGAATCGGATAGGTCAAAC |
| YNL095C | BB3143 | PR-22034 | GGTTTAAUTCAAAGGTTCATCTGTACTTTCAG |
| YOL060C | BB3145 | PR-22036 | GGTTTAAUTCATCTTTTTATGGTCGACGAGGAG |
| YOL092W | BB3147 | PR-22038 | GGTTTAAUTCAATTCAGTATAAATTTTTTATTTC |
| YOR092W | BB3149 | PR-22040 | GGTTTAAUTCATAATTTCATTTGAACTTTGATG |
| YOR271C | BB3151 | PR-22042 | GGTTTAAUTCAAATACCTCTGTTAAAATAGACC |
| YOR307C | BB3152 | PR-22043 | GGTTTAAUTCATGCCTGACGTCCATCTTTTTTGG |
| YPL189W | BB3156 | PR-22047 | GGTTTAAUTCAACATTTCAAGTTGATGCCATG |
| YPL244C | BB3157 | PR-22048 | GGTTTAAUTCACGCAGATTTTGCCTTCGG |
| YPL264C | BB3158 | PR-22049 | GGTTTAAUTCAATCCTCCAAATCATCTAATTC |
| YPR201W | BB3160 | PR-22051 | GGTTTAAUTCAATTTCTATTGTTCCATATATAATA |
| yEGFP | BB2959 | PR-22052 | GGTTTAAUTCATTTGTACAATTCATCCATAC |

## Table S3

Sequencing primers

| Transporter | Primer name | Primer ID | Sequence primer |
| --- | --- | --- | --- |
| Universal sequencing primer R |  | PR-22259 | AAGGCGATTAAGTTGGGT |
| Universal sequencing primer F |  | PR-22260 | GTGTTCTTGAGGCTGGTTTA |
| YAL022C | Primer 1 | PR-22261 | TGTGTTGTTAGGGTCTCTGC |
| YBL020W | Primer 1 | PR-22262 | CAGGCCCAAATACAATGA |
| YBR235W | Primer 1 | PR-22263 | ACAGCTGGTATATTCGCGGG |
|  | Primer 2 | PR-22264 | GAGTCCTCACTCTCGCATGG |
|  | Primer 3 | PR-22265 | CCAAGGACGAGCTGGAACTT |
|  | Primer 4 | PR-22363 | CACCCATGAATATAACCC |
| YHR032W | Primer 1 | PR-22578 | ATTCCGGAGAGAGAACTA |
| YDL231C | Primer 1 | PR-22979 | AGTGGATATGATGGCAGA |
|  | Primer 2 | PR-22980 | GGAACTGTTGAATGACGA |
|  | Primer 3 | PR-22981 | ATCGAGCTTCACTGCATT |
| YDR338C | Primer 1 | PR-22865 | GAGGCACTAGCAGAATTG |
|  | Primer 2 | PR-22931 | CCCCAGCATTTTCTTCCA |
| YGL084C | Primer 1 | PR-22932 | CCGCTCATGGTGTCAATTC |
| YGL140C | Primer 1 | PR-22866 | AGGCAAGCTATTGAAGATGA |
|  | Primer 2 | PR-22867 | GAAAATGTCTCGTAGTTGGT |
|  | Primer 3 | PR-22868 | GAGTCCTAGTGATGAAATG |
|  | Primer 4 | PR-22869 | CTGCTGTCAACCAAACCAA |
|  | Primer 5 | PR-23776 | ACGAAACAGGATCTCTAC |
| YNL095C | Primer 1 | PR-23493 | CAACAATAAGTACACCGC |
| YOL060C | Primer 1 | PR-23494 | GATTAGAATTGGATGGAGAC |
|  | Primer 2 | PR-23495 | TATGAGACAACAACCGGG |
| YOR092W | Primer 1 | PR-23503 | TTCTTCTTCTTCATCCAACC |
| YPL189W | Primer 1 | PR-23550 | GGATCAATGGTATAAGGG |
|  | Primer 2 | PR-23736 | TCGTTCCTTTTCCTCCAGA |

## Table S4

Plasmids generated in this study

| Transporter | Plasmid no | Genomic template |
| --- | --- | --- |
| yEGFP | pCfB8156 |  |
| YAL022C | pCfB8150 | BY4741 |
| YBL020W | pCfB8151 | CEN.PK |
| YBR147W | pCfB8152 | BY4741 |
| YBR235W | pCfB8153 | CEN.PK |
| YBR287W | pCfB8154 | BY4741 |
| YCL002C | pCfB8155 | BY4741 |
| YCR075C | pCFB8455 | CEN.PK |
| YEL004W | pCFB8456 | BY4741 |
| YER039C | pCFB8457 | CEN.PK |
| YHR032W | pCfB8360 | BY4741 |
| YDL231C | pCfB8704 | BY4741 |
| YDR338C | pCfB8405 | BY4741 |
| YDR352W | pCfB8406 | BY4741 |
| YDR438W | pCfB8407 | BY4741 |
| YGL084C | pCfB8408 | BY4741 |
| YGL140C |  |  |
| YJL193W | pCfB8410 | BY4741 |
| YLR152C | pCfB8511 | BY4741 |
| YML018C | pCfB8586 | BY4741 |
| YML038C | pCfB8587 | BY4741 |
| YMR034C | pCfB8588 | BY4741 |
| YMR253C | pCfB8589 | BY4741 |
| YNL095C | pCfB8707 | BY4741 |
| YOL060C | pCfB8728 | BY4741 |
| YOL092W | pCfB8709 | BY4741 |
| YOR092W | pCfB8711 | BY4741 |
| YOR271C | pCfB8713 | BY4741 |
| YOR307C | pCfB8590 | BY4741 |
| YPL189W | pCfB8738 | BY4741 |
| YPL244C | pCfB8591 | BY4741 |
| YPL264C | pCfB8592 | BY4741 |
| YPR201W | pCfB8715 | BY4741 |

## Table S5

Calculations of LC50. The growth experiments were carried out in triplicates

2-methylpyrazine

| Concentration (mM) | 0 | 0.02 | 2 | 20 | 50 | 200 | 750 | 1000 |
| --- | --- | --- | --- | --- | --- | --- | --- | --- |
| Max OD measured | 25.3 | 29.0 | 27.4 | 27.4 | 23.3 | 9.9 | 0.22 | 0.42 |

2-benzoxazolol

| Concentration (mM) | 0 | 0.02 | 2 | 4 | 8 | 12 | 16 | 20 |
| --- | --- | --- | --- | --- | --- | --- | --- | --- |
| Max OD measured | 25.9 | 29.1 | 25.3 | 22.4 | 9.7 | 8.5 | 2.0 | 5.4 |

acrylic acid

| Concentration (mM) | 0 | 0.02 | 0.2 | 2 | 20 |
| --- | --- | --- | --- | --- | --- |
| Max OD measured | 25.0 | 27.5 | 25.6 | 17.5 | 6.8 |

Cefadroxil

| Concentration (mM) | 0 | 0.02 | 2 | 20 | 28 |
| --- | --- | --- | --- | --- | --- |
| Max OD measured | 26.8 | 28.7 | 27.8 | 26.1 | 23.7 |

2,5-furandicarboxylic acid

| Concentration (mM) | 0 | 0.02 | 2 | 4 | 8 | 12 | 16 | 20 |
| --- | --- | --- | --- | --- | --- | --- | --- | --- |
| Max OD measured | 25.3 | 27.8 | 26.5 | 29.8 | 23.6 | 17.2 | 10.3 | 1.9 |

Sn-glycero-3-phosphocholine

| Concentration (mM) | 0 | 0.2 | 2 | 20 | 50 | 100 | 300 | 787 |
| --- | --- | --- | --- | --- | --- | --- | --- | --- |
| Max OD measured | 26.3 | 26.7 | 27.1 | 29.3 | 26.5 | 25.0 | 19.7 | 14.7 |

## Table S5

Direction and mode of transport for the identified transporters

|  | **FDC** | **BXZ** | **CFX** | **MPZ** | **GPC** | **ACA** |
| --- | --- | --- | --- | --- | --- | --- |
| **Thi74** | Imp. (equil.) | Exp. | Imp. (equil.) |  | Imp. (equil.) |  |
| **YLR152C** |  |  | Exp. | Imp. (equil.) | Exp. | Imp. (conc.) |
| **YML018C** |  |  | Imp. (equil.) |  | Imp. (equil.) |  |
| **Mam3** |  |  | Imp. (equil.) |  |  |  |
| **Rtc2** |  | Imp. (conc.) |  |  |  |  |
| **Ecm3** |  | Imp. (conc.) |  |  |  |  |
| **YNL095C** | Imp. (equil.) |  |  | Imp. (equil.) |  | Imp. (conc.) |
| **Sly41** | Imp. (equil.) |  |  |  |  |  |
| **Hut1** | Exp. |  |  |  |  |  |
| **Ypq2** |  |  |  | Imp. (equil.) | Exp. | Imp. (conc.) |
| **Yea4** |  |  |  |  | Exp. |  |
| **Hvg1** |  |  |  |  | Imp. (equil.) |  |
| **Gup1** |  |  |  |  | Imp. (equil.) |  |
| **Erc1** |  |  |  |  | Imp. (equil.) |  |
| **Ymd8** |  |  |  |  | Imp. (equil.) |  |
| **Rch1** |  |  |  |  | Exp. |  |
| **YMR253C** |  |  |  |  | Exp. |  |
| **Fsf1** |  |  |  |  | Exp. | Imp. (conc.) |
| **YDR338C** |  |  |  |  |  |  |
| **Ypq1** |  |  |  |  |  | Imp. (conc.) |

## Table S6

TCDB classification of identified transporters

| **Protein** | **TCID** | **Superfamily ID** | **Transporter Superfamily** | **Class ID** | **Transporter Class** |
| --- | --- | --- | --- | --- | --- |
| Mam3 | unclassified | 1.A.112* | The Cyclin M Mg2+ Exporter (CNNM) Family | 1.A | α-Type Channels |
| Hvg1 | 2.A.7.9 | 2.A.7 | The Drug/Metabolite Transporter (DMT) Superfamily | 2.A | Porters (uniporters, symporters, antiporters) |
| Sly41 | 2.A.7.9.4 | 2.A.7 | The Drug/Metabolite Transporter (DMT) Superfamily | 2.A | Porters (uniporters, symporters, antiporters) |
| Ymd8 | 2.A.7.9.14 | 2.A.7 | The Drug/Metabolite Transporter (DMT) Superfamily | 2.A | Porters (uniporters, symporters, antiporters) |
| Yea4 | 2.A.7.10.5 | 2.A.7 | The Drug/Metabolite Transporter (DMT) Superfamily | 2.A | Porters (uniporters, symporters, antiporters) |
| Hut1 | 2.A.7.11.6 | 2.A.7 | The Drug/Metabolite Transporter (DMT) Superfamily | 2.A | Porters (uniporters, symporters, antiporters) |
| Thi74 | 2.A.7.24.1 | 2.A.7 | The Drug/Metabolite Transporter (DMT) Superfamily | 2.A | Porters (uniporters, symporters, antiporters) |
| Yml018c | 2.A.7.24.5 | 2.A.7 | The Drug/Metabolite Transporter (DMT) Superfamily | 2.A | Porters (uniporters, symporters, antiporters) |
| Ymr253c | unclassified | 2.A.7* | The Drug/Metabolite Transporter (DMT) Superfamily | 2.A | Porters (uniporters, symporters, antiporters) |
| Rch1 | 2.A.28.3.7 | 2.A.28 | The Bile Acid:Na^+^ Symporter (BASS) Family | 2.A | Porters (uniporters, symporters, antiporters) |
| Ypq1 | 2.A.43.2.3 | 2.A.43 | The Lysosomal Cystine Transporter (LCT) Family | 2.A | Porters (uniporters, symporters, antiporters) |
| Rtc2 | 2.A.43.2.7 | 2.A.43 | The Lysosomal Cystine Transporter (LCT) Family | 2.A | Porters (uniporters, symporters, antiporters) |
| Ypq2 | 2.A.43.2.8 | 2.A.43 | The Lysosomal Cystine Transporter (LCT) Family | 2.A | Porters (uniporters, symporters, antiporters) |
| Gup1 | 2.A.50.1.1 | 2.A.50 | The Glycerol Uptake (GUP) or Membrane-bound Acyl Transferase (MBOAT) Family | 2.A | Porters (uniporters, symporters, antiporters) |
| Fsf1 | 2.A.54.1.4 | 2.A.54 | The Sideroflexin (SFXN) Family (formerly the Mitochondrial Tricarboxylate Carrier (MTC) Family) | 2.A | Porters (uniporters, symporters, antiporters) |
| Ydr338c | 2.A.66.1 | 2.A.66 | The Multidrug/Oligosaccharidyl-lipid/Polysaccharide (MOP) Flippase Superfamily | 2.A | Porters (uniporters, symporters, antiporters) |
| Erc1 | 2.A.66.1.5 | 2.A.66 | The Multidrug/Oligosaccharidyl-lipid/Polysaccharide (MOP) Flippase Superfamily | 2.A | Porters (uniporters, symporters, antiporters) |
| Ylr152c | 2.A.69.2.6 | 2.A.69 | The Auxin Efflux Carrier (AEC) Family | 2.A | Porters (uniporters, symporters, antiporters) |
| Ecm3 | unclassified | 2.A.69* | The Auxin Efflux Carrier (AEC) Family | 2.A | Porters (uniporters, symporters, antiporters) |
| Ynl095c | unclassified | 2.A.69* | The Auxin Efflux Carrier (AEC) Family | 2.A | Porters (uniporters, symporters, antiporters) |

*Transporter superfamily identified by blastp search for closest homolog in TCDB, using standard blast parameters.

TCID for all remaining transporters found by searching available protein databases (TCDB (tcdb.org), Uniprot (uniprot.org) or SGD (yeastgenome.org))

## Figure S1


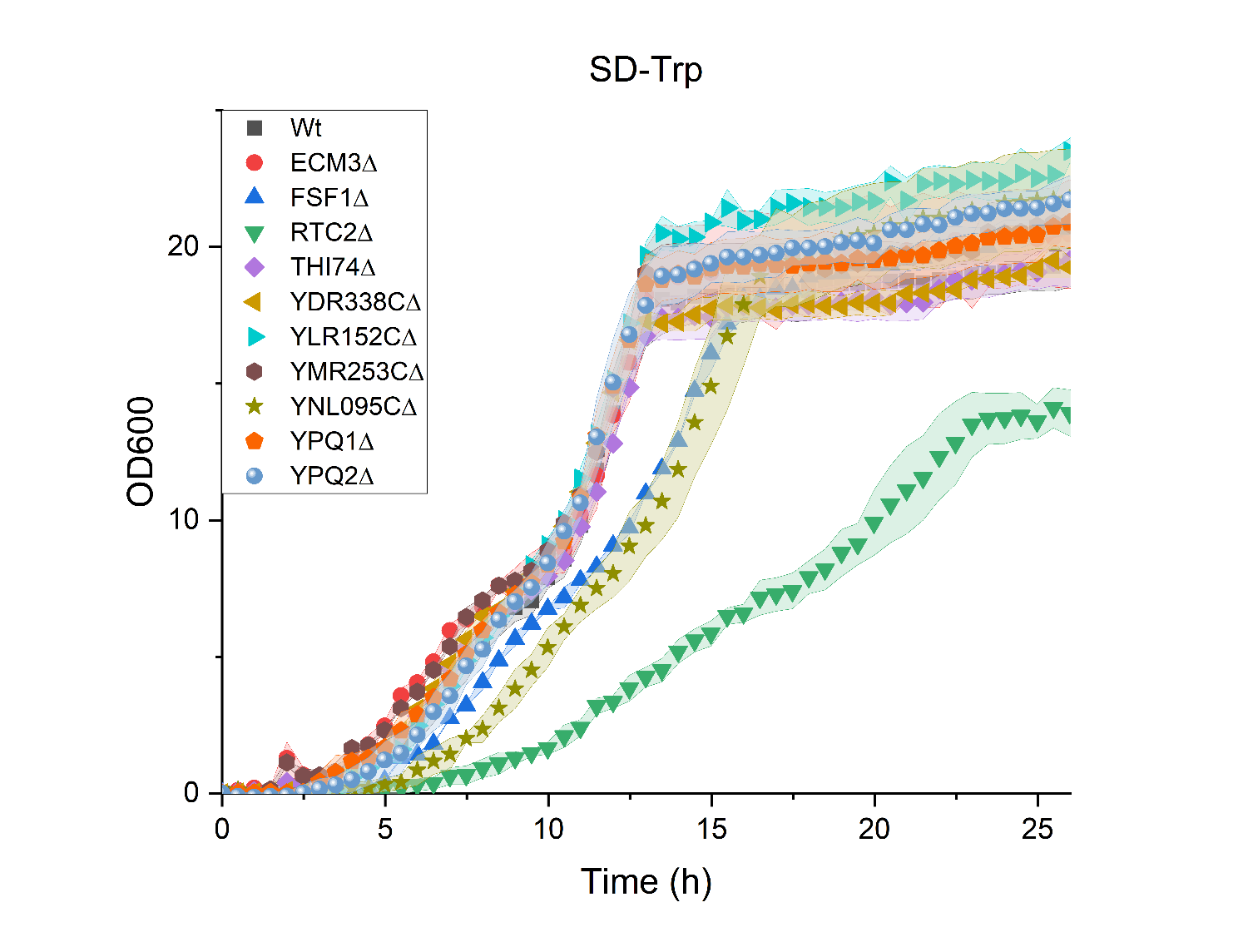
Figure S1: Growth in SD-Trp

Growth assay of strains harboring deletions of transporters genes identified in the screening effort. The strains are grown in SD-Trp

## Figure S2


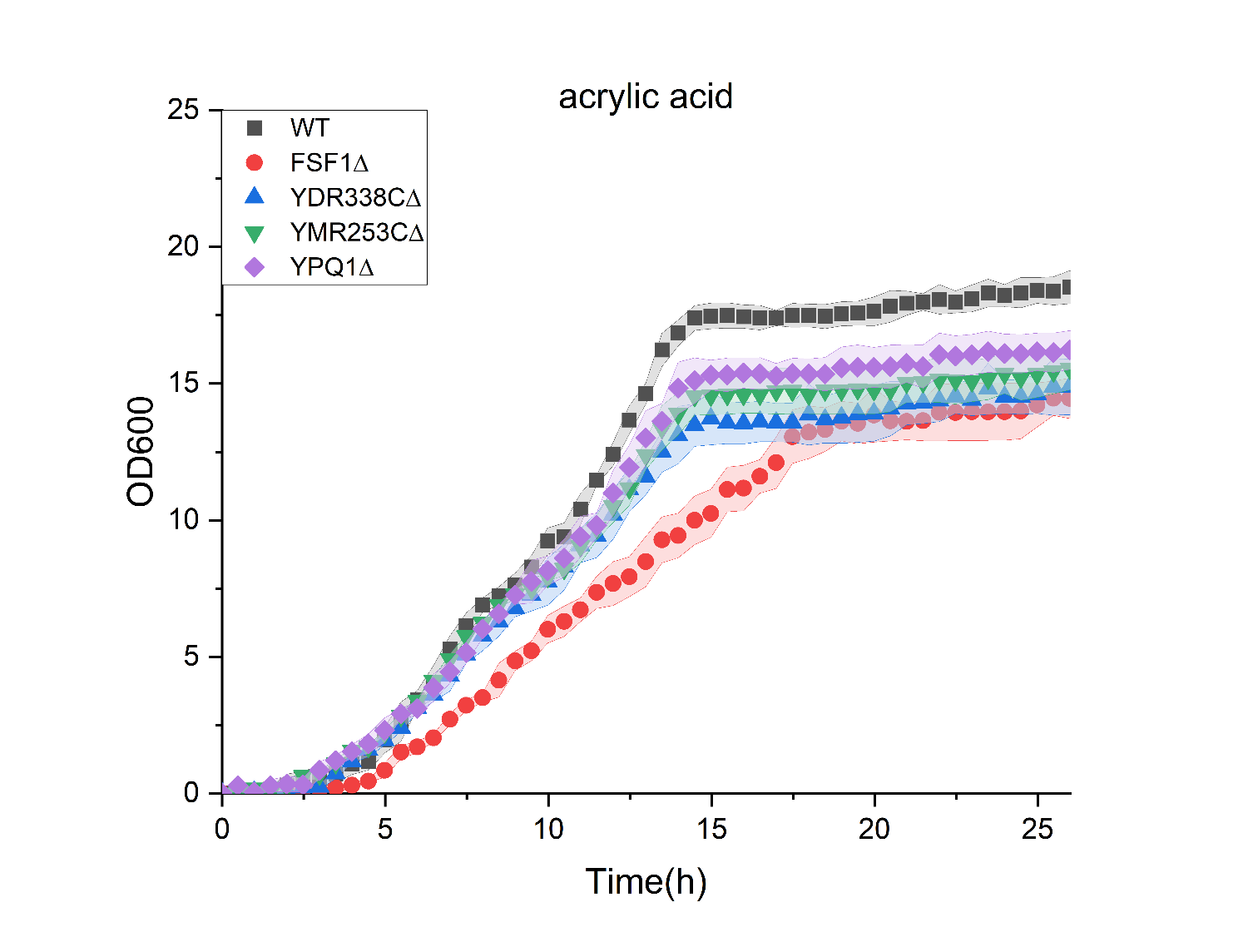


Figure S2: Strains grown in SD-Trp supplemented with acrylic acid

Growth assay of strains harboring deletions of transporters genes identified in the screening effort as transporter proteins having transport activity towards acrylic acid. The assay was conducted with 2.45 mM acrylic acid.

## Figure S3


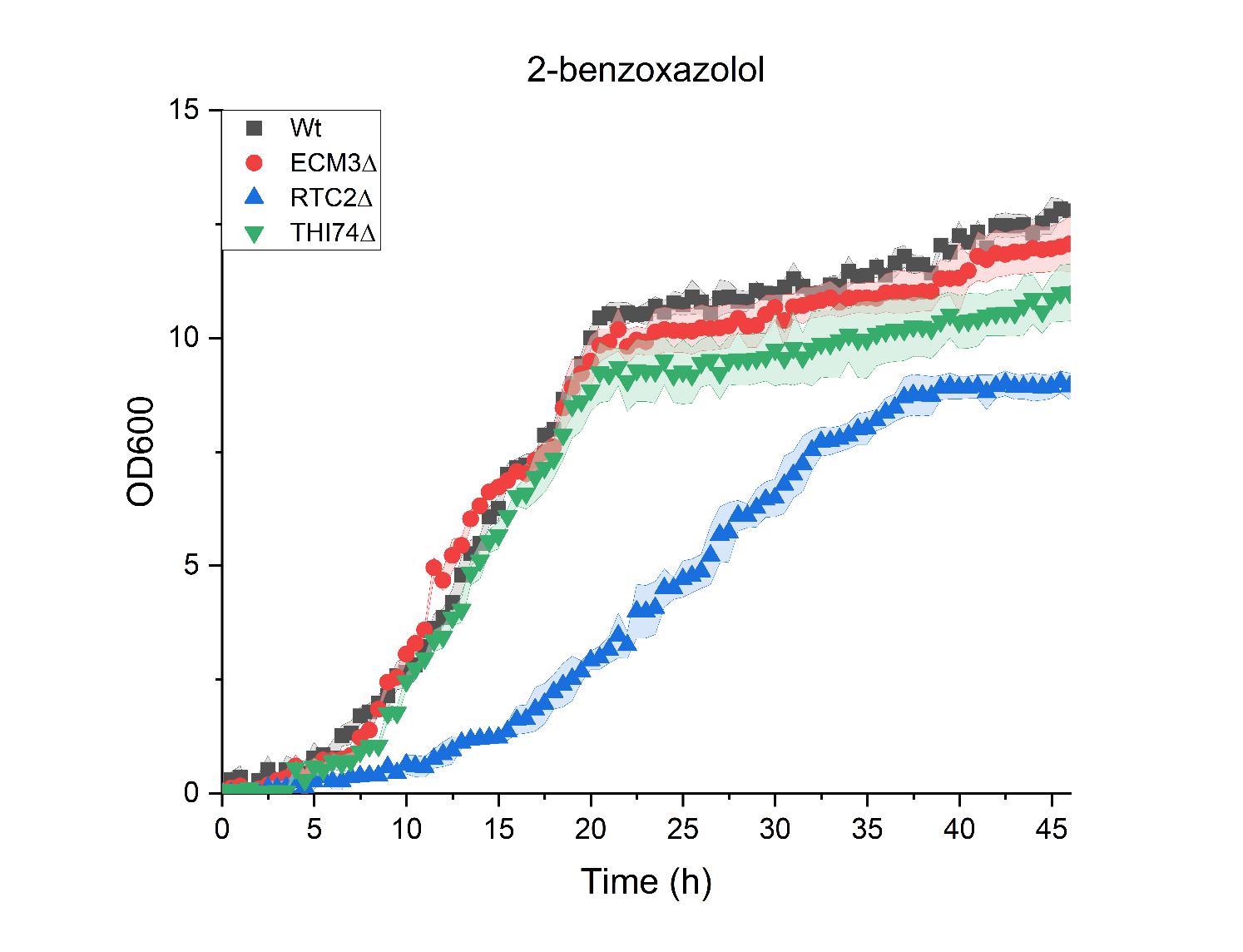


Figure S3: Strains grown in SD-Trp supplemented with 2-benzoxazolol

Growth assay of strains harboring deletions of transporters genes identified in the screening effort as transporter proteins having transport activity towards 2-benzoxazolol. The assay was conducted with 6.44 mM 2-benzoxazolol.

## Figure S4


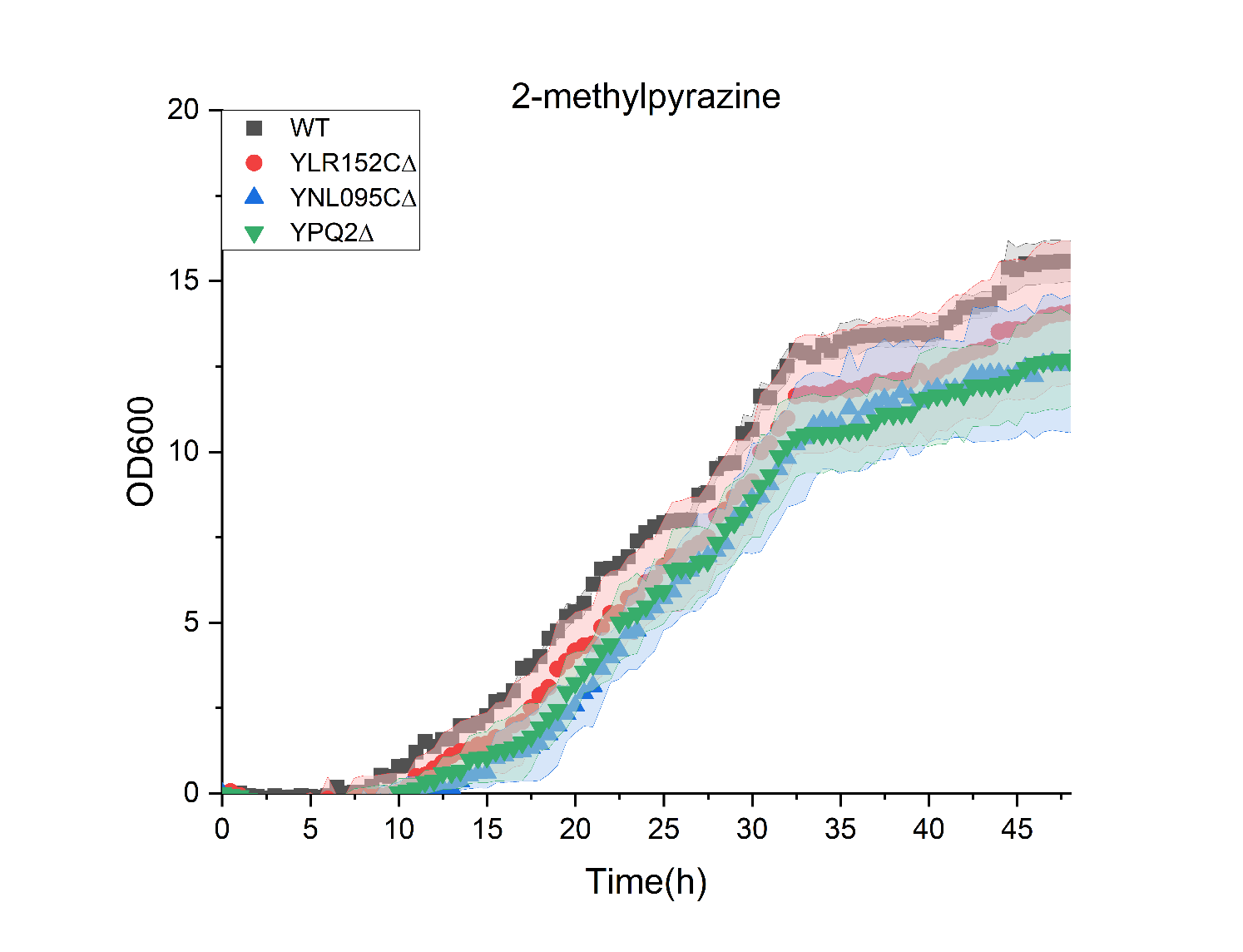


Figure S4: Strains grown in SD-Trp supplemented with 2-benzoxazolol

Growth assay of strains harboring deletions of transporters genes identified in the screening effert as transporter proteins having transport activity towards methylpyrazine. The assay was conducted with 155.15 mM methylpyrazine
